# Magnetic Hyperthermia via Zn_0.2_Mn_0.8_Fe_2_O_4_ Oleic Acid Nanoparticles Enhances Chemotherapy Efficacy in a Lewis Lung Carcinoma Model

**DOI:** 10.3390/pharmaceutics18081021

**Published:** 2026-08-17

**Authors:** Denis E. Yakobson, Mikhail N. Zharkov, Oleg A. Kulikov, Vasilisa I. Kulikova, Vladislav S. Bobrov, Aleksey O. Makarov, Ekaterina P. Brodovskaya, Larisa A. Balykova, Ran Yan, Nikolay A. Pyataev

**Affiliations:** 1 Federal Center for Biotechnology and Medicine Advancement, National Research Ogarev Mordovia State University (MRSU), Bolshevistskaya Str. 68, 430005 Saransk, Russia; mikhail.zharkov.92@mail.ru (M.N.Z.); oleg-kulikov-84@mail.ru (O.A.K.); shlyapkina.98@mail.ru (V.I.K.); vlad_bobrov_02@list.ru (V.S.B.); makar06.rus@mail.ru (A.O.M.); ekbrodovskaya@gmail.com (E.P.B.); larisabalykova@yandex.ru (L.A.B.); 2 Key Laboratory of Biomedical Functional Materials, School of Science, China Pharmaceutical University, 639 Longmian Avenue, Nanjing 211198, China; frankyan@cpu.edu.cn

**Keywords:** chemotherapy, magnetic hyperthermia, zinc-manganese ferrite, oleic acid, cisplatin, Lewis lung carcinoma

## Abstract

**Background/Objectives**: Combining chemotherapy with local magnetic hyperthermia (MHT) is promising because heating tumor tissue can increase cell damage, sensitize cells to cytostatic drugs, impair DNA repair, and change tumor microenvironment permeability. This creates conditions for enhancing the antitumor efficacy of chemotherapy while potentially reducing systemic toxicity. The aim of this study was to evaluate the efficacy of MHT with Zn_0.2_Mn_0.8_Fe_2_O_4_@OA nanoparticles alone and in combination with cisplatin in a Lewis lung carcinoma model. **Methods**: Four types of magnetic nanoparticles were synthesized and characterized: Fe_3_O_4_ and Zn_0.2_Mn_0.8_Fe_2_O_4_ coated with oleic acid (OA) or SiO_2_-NH_2_. Their physicochemical and magnetothermal properties, cytotoxicity, reactive oxygen species generation, and biodegradation in vivo were evaluated. Antitumor efficacy was studied in C57Bl/6 mice with LLC tumors after intratumoral administration of nanoparticles and two MHT sessions (100 kHz, 8 kA/m, 30 min). In combination therapy, ZnMn@OA and cisplatin at doses of 9 or 18 mg/kg were used. **Results/Conclusions**: Zn_0.2_Mn_0.8_Fe_2_O_4_@OA combined efficient heating, biodegradation, and the most pronounced effect among the MHT-alone groups, although MHT without chemotherapy did not provide sustained inhibition of tumor growth or a significant increase in survival. The combination of Zn_0.2_Mn_0.8_Fe_2_O_4_@OA-MHT with cisplatin 9 mg/kg produced the best therapeutic outcome: median survival increased significantly by two fold compared with the control group and by 1.8-fold compared with the chemotherapy-alone group at the comparable cisplatin dose. This regimen also stabilized body weight, reduced systemic toxicity, and restored RBC, HGB, and HCT parameters to the level of healthy animals by day 7 of the experiment. These data confirm the potential of MHT as a chemosensitizing approach that improves the efficacy and tolerability of cisplatin therapy.

## 1. Introduction

Therapy of malignant neoplasms remains one of the pressing challenges of modern medicine. Chemotherapy remains an important component of modern antitumor treatment, but its use is limited by systemic toxicity, insufficient selectivity of action, and the development of drug resistance in tumor cells [1,2]. In this regard, a promising approach is the combination of chemotherapy with local physical methods of exposure capable of enhancing the effect on tumor tissues and reducing the toxic burden on the body [2,3,4].

Such approaches include magnetic hyperthermia (MHT), based on the local administration of magnetic nanoparticles (MNPs) into tumor tissue followed by exposure to an alternating magnetic field (AMF), which leads to heating of the particles and an increase in temperature in the tumor [5]. The advantages of MHT include the locality and controllability of the effect, minimal invasiveness, the use of nonionizing fields, the ability to heat deeply located tissues, and limited damage to surrounding healthy structures [6].

MHT can be used as a standalone method [7,8]. However, its combination with chemotherapy is of greatest interest [9,10]. A local increase in temperature can cause thermal damage to tumor cells [11], enhance oxidative stress [12], disrupt the structure of proteins, cell membranes, and organelles [13], as well as affect DNA repair processes [13,14], increasing the sensitivity of tumor cells to the action of the chemotherapeutic agent [15,16]. Moreover, hyperthermia can alter vascular permeability and the tumor microenvironment, promoting more effective penetration of chemotherapeutic agents into tumor tissue [17]. This creates conditions for enhancing chemotherapy’s antitumor effect and potentially reducing the cytostatic dose without compromising efficacy.

The efficacy of magnetic hyperthermia is determined by a set of properties of pharmaceutical compositions based on magnetic nanoparticles, including size, shape, magnetic core composition, type of surface coating, colloidal stability, biocompatibility, and specific absorption rate (SAR), reflecting the ability of MNPs to convert the energy of an alternating magnetic field into heat [10,18,19].

Iron oxide nanoparticles (IONPs), primarily magnetite Fe_3_O_4_, which have proven biocompatibility, are the most extensively studied nanoparticles for magnetic hyperthermia [20]. However, studies show that magnetite is not always the optimal material in terms of SAR [10]. SAR reflects the amount of heat generated by magnetic nanoparticles per unit time and per unit mass of magnetic material under exposure to an alternating magnetic field and is typically expressed in W/g [21]. An alternative may be ferrite nanoparticles MeFe_2_O_4_ (Me = Zn^2+^, Mn^2+^, Ni^2+^, Cu^2+^, Co^2+^), in which the magnetic properties can be purposefully optimized by incorporating other metals into their composition [22]. In particular, zinc–manganese ferrite Zn_0.2_Mn_0.8_Fe_2_O_4_ demonstrated greater heating efficiency under AMF exposure due to the optimal combination of cationic composition and nonlinear magnetic response [23,24,25].

Another important aspect is the nanoparticle coating, which determines their colloidal stability, interaction with the biological environment, and subsequent biodegradation. For example, the clinically used NanoTherm AS1 system (MagForce AG) is a suspension of magnetite nanoparticles (112 mg Fe/mL) with a shell based on amino-functionalized silica (SiO_2_-NH_2_) and is one of the best-known examples of the translation of magnetic hyperthermia into clinical practice [26,27]. Such a shell ensures the colloidal stability of the particles, their efficient retention in tissue, and good heating upon AMF exposure. At the same time, the presence of an inorganic SiO_2_-NH_2_ shell may limit the biodegradation of such particles and contribute to their long-term persistence in tissues. In individual clinical cases, the need to remove residual nanomaterial has been described [28,29]. Therefore, the use of biocompatible organic coatings is promising, particularly those based on oleic acid, which can effectively stabilize MNPs [30,31,32] and potentially promote MNP biodegradation after local administration.

Despite the promise of magnetic hyperthermia and the active study of MNPs, a number of questions remain open. Most studies focus on the physicochemical characterization of nanoparticles, their magnetothermal properties, and in vitro effects [9,33,34,35], whereas data on the actual antitumor efficacy of MHT in vivo under safe AMF parameters [36,37] remain insufficient. Moreover, the limits of the therapeutic applicability of MHT as a standalone treatment modality and its ability to enhance the effect of chemotherapy in the treatment of solid tumors have not been sufficiently defined.

This study aimed to evaluate the therapeutic potential and limits of magnetic hyperthermia alone and combined with cisplatin under permissible magnetic field parameters in a Lewis lung carcinoma model.

## 2. Materials and Methods

### 2.1. Reagents

Deionized water Nova U (Innova Bio Meditech, Shanghai, China); iron(II) chloride tetrahydrate (FeCl_2_·4H_2_O), iron(III) chloride hexahydrate (FeCl_3_·6H_2_O), zinc chloride (ZnCl_2_), manganese(II) chloride tetrahydrate (MnCl_2_·4H_2_O), oleic acid (C_18_H_34_O_2_), ammonium hydroxide (25%NH_4_OH), sodium hydroxide (NaOH), hydrochloric acid (37% HCl), (Vekton, Saint Petersburg, Russia); N-(3-(Trimethoxysilyl)propyl)ethylenediamine (Sigma-Aldrich, St. Louis, MO, USA); Zoletil (Virbac, Carros, France); Rometar (Bioveta, Ivanovice na Hané, Czech Republic); cisplatin (Pharmasintez-Nord JSC, Saint Petersburg, Russia). All reagents used in this study were of at least chemically pure grade.

### 2.2. Synthesis of Iron Oxide and Zn_0.2_Mn_0.8_Fe_2_O_4_ Nanoparticles

Magnetite (Fe_3_O_4_) and zinc–manganese ferrite (Zn_0.2_Mn_0.8_Fe_2_O_4_) nanoparticles were synthesized by coprecipitation in an alkaline ammonia medium [25,38]. All synthesis steps were performed under an argon atmosphere.

Synthesis of Fe_3_O_4_: Iron salts (1.35 g FeCl_2_·4H_2_O; 3.5 g FeCl_3_·6H_2_O) were dissolved in 50 mL of deionized water preheated to 100 °C. Then, 9 mL of 25%NH_4_OH was added to the resulting solution in a single portion under vigorous stirring. The resulting IONPs suspension was incubated with stirring for 1 h at 90 °C.

Synthesis of Zn_0.2_Mn_0.8_Fe_2_O_4_: Weighed portions of metal salts (0.1768 g ZnCl_2_; 1.03 g MnCl_2_·4H_2_O, 3.5 g FeCl_3_·6H_2_O) were dissolved in 35 mL of deionized water heated to 120 °C. The resulting salt solution was rapidly poured into 50 mL of a heated NaOH solution (2.5 g). The suspension was incubated with stirring for 2 h at 180 °C.

Purification of IONPs and zinc–manganese ferrite nanoparticles was performed by magnetic decantation (10 cycles) using a mixture of deionized water and ethanol (volume ratio 4:1) until a neutral pH value of the supernatant was reached. After every two washing cycles, the magnetic particles were subjected to ultrasonication (power 500 W (Noise isolation chamber 600 W, Hangzhou, China), duration 10 min).

#### Stabilization of Nanoparticles

Stabilization with oleic acid/sodium oleate (Fe_3_O_4_@OA, Zn_0.2_Mn_0.8_Fe_2_O_4_@OA) was performed according to our previously described optimized method [38].

Briefly, the stabilizer solution was prepared by dissolving 0.16 g NaOH in a mixture of deionized water (2 mL) and ethanol (3 mL), followed by the addition of 1.75 mL of oleic acid under stirring. The freshly prepared mixture was added to the nanoparticles and incubated for 2 h at 150 °C under vigorous stirring, with ethanol evaporating during heating. Every 30 min, the suspension was subjected to ultrasonic (US) treatment (10 min, 500 W). Large aggregates and unstabilized particles were removed by centrifugation (12,000 rpm, 15 min), and the resulting supernatant was concentrated by evaporation to 154 mg/mL with respect to the magnetic material.

Nanoparticle coating was performed according to a modified method designed to mimic the approach used in the development of NanoTherm^®^ AS1 (MagForce AG, Berlin, Germany) [39]. The SiO_2_-NH_2_ shell was formed on the nanoparticles by hydrolysis of N-(3-(Trimethoxysilyl)propyl)ethylenediamine (Fe_3_O_4_@SiO_2_–NH_2_, Zn_0.2_Mn_0.8_Fe_2_O_4_@SiO_2_–NH_2_).

Briefly, 520 µL of the organosilicon precursor and 550 µL of 37% HCl were simultaneously added dropwise to the purified nanoparticles under vigorous stirring (900 rpm, 25 °C) for 30 min to maintain an acidic medium (pH 2.5–3.0). The mixture was subjected to ultrasonication (150 W, 24 h). Reaction by-products were removed by dialysis (cellulose membrane, 12–14 kDa) for 72 h. To remove large aggregates, the purified nanoparticle suspension was centrifuged (2000 rpm, 10 min), and the resulting supernatant was then concentrated by evaporation to 154 mg/mL with respect to the magnetic material.

As a result, four highly concentrated suspensions of magnetic nanoparticles were obtained: Fe_3_O_4_@OA (hereafter Fe@OA), Zn_0.2_Mn_0.8_Fe_2_O_4_@OA (hereafter ZnMn@OA), Fe_3_O_4_@SiO_2_–NH_2_ (hereafter Fe@SiO_2_), Zn_0.2_Mn_0.8_Fe_2_O_4_@SiO_2_–NH_2_ (hereafter ZnMn@SiO_2_).

### 2.3. Characterization

Five independent batches of each nanoparticle type were synthesized. Each batch was characterized in terms of its main physicochemical parameters. The hydrodynamic size of the synthesized MNPs was measured by dynamic light scattering (DLS) using a NANO-flex instrument (working range 0.8–5500 nm), and the electrokinetic potential (ζ-potential) was determined using a STABINO analyzer (Microtrac, Haan, Germany). The polydispersity index (PDI) was calculated according to the formula: PDI = σ^2/^d^2^, where d^2^ is the weighted mean of the size distribution; σ^2^ is the weighted mean square deviation. The morphology and size of MNPs were studied by transmission electron microscopy (TEM) using a Tecnai Osiris microscope (FEI, Hillsboro, OR, USA). The phase composition of the samples was determined using a PANalytical Empyrean X-ray diffractometer (Almelo, The Netherlands) with Cu Kα radiation (λ = 1.5406 Å) and a PIXcel 3D linear detector. Phase identification was performed based on the positions of the main reflections characteristic of the cubic spinel structure with the Fd-3m space group. Quantitative determination of iron in the obtained magnetic suspensions was performed by a photocolorimetric method using a Shimadzu UV-2600 spectrophotometer (Kyoto, Japan). The method is based on measuring the optical absorption of the ferric iron complex with sulfosalicylic acid formed in an alkaline medium (pH 9–12) at λ = 430 nm [40]. The concentration was calculated from the calibration curve (R^2^ = 0.9996, limit of detection 0.1 mg/L), after which it was recalculated to the total mass of the corresponding ferrites taking into account their stoichiometry. In all experiments, the concentration of the suspensions was expressed in mg/mL with respect to the magnetic material. The magnetization of the samples was studied using an EZ11 vibrating sample magnetometer (Microsense Inc., Lowell, MA, USA) at 25 °C. To evaluate the magnetocaloric properties of the suspensions (154 mg/mL, volume 0.5 mL), a custom induction setup generating an alternating magnetic field (AMF) with a frequency of f = 100 kHz and a maximum field strength of H = 8 kA/m was used [41]. The samples were placed in the center of a thermally insulated, liquid-cooled solenoid and exposed to the alternating magnetic field for 10 min. The heating efficiency of the MNPs was evaluated using the specific absorption rate (SAR), expressed in W/g of magnetic material. SAR was calculated from the initial linear region of the temperature–time curve according to the following Equation (1) [42]:

(1) SAR = C × (M/m) × (dT/dt),

where C is the specific heat capacity of the liquid, M is the mass of the suspension, m is the mass of magnetic material in the sample, and dT/dt is the initial rate of temperature increase determined from the linear region of the heating curve. The specific heat capacity of water was taken as 4190 J × kg^−1^ × K^−1^.

### 2.4. Cell Lines and Culture

The L929 mouse fibroblast cell culture was obtained from the Tissue Culture Collection of the N. F. Gamaleya Research Institute of Epidemiology and Microbiology of the Ministry of Health of the Russian Federation. The LLC1 Lewis lung carcinoma cell culture was obtained from “Algimed LLC” (Moscow, Russia). Cells were cultured in DMEM medium (PanEco, Moscow, Russia) supplemented with 10% fetal bovine serum (INTL KANG, Hong Kong, China) and 1% penicillin–streptomycin antibiotic solution (Servicebio, Wuhan, China) under standard conditions at 37 °C and 5% CO_2_. Upon reaching 80–90% confluence, the cells were passaged into 96-well plates (SPL, Pocheon-si, Korea) for further studies.

#### 2.4.1. Cytotoxicity Assay

Nanoparticle cytotoxicity was studied using the MTT assay. For this purpose, cells were seeded into a 96-well plate at a density of 5 × 10^3^ cells/well. Nanoparticle solutions at concentrations of 0–1000 µg/mL were added to the cells. After 24 h of incubation under standard conditions, the cells were washed three times with PBS solution (Servicebio, Wuhan, China). Hanks’ solution (PanEco, Russia) and 10 µL of MTT (3-(4,5-dimethylthiazol-2-yl)-2,5-diphenyltetrazolium bromide) at a concentration of 5 mg/mL were then added to the cells. After 3.5 h, the resulting formazan crystals were dissolved with DMSO solution. Optical density was measured using a Varioskan Lux plate reader (Thermo Scientific, Waltham, MA, USA) at a wavelength of 570 nm and 650 nm. Cell viability was expressed as a percentage relative to intact control cells incubated in culture medium.

#### 2.4.2. ROS Detection

The level of reactive oxygen species (ROS) in the LLC1 cell culture was assessed using the fluorescent dye H_2_DCFDA (Lumiprobe, Moscow, Russia) [43]. Cells were seeded into a 96-well plate at a density of 1 × 10^4^ cells/well. After 24 h of incubation, the cells were washed three times with Hanks’ solution. The prepared H_2_DCFDA working solution (Lumiprobe) was added to all wells at a concentration of 10 µM (5 µg/mL), 100 µL per well. The cells were incubated for 45 min in the dark in an incubator. Then, the H_2_DCFDA solution was removed, and medium containing the studied MNPs at concentrations of 15.625–125 µg/mL was added. A 0.8% hydrogen peroxide (H_2_O_2_) solution was added as a positive control. Under the action of intracellular ROS, H_2_DCFDA is oxidized to form the fluorescent product 2′,7′-dichlorofluorescein. After 24 h of incubation, the fluorescence intensity of 2′,7′-dichlorofluorescein, expressed in relative fluorescence units (RFU), was determined using a Varioskan Lux plate reader (Thermo Scientific, Waltham, MA, USA) at an excitation wavelength of 485 nm and an emission wavelength of 535 nm. Intact cells were used as the control. The higher the fluorescence intensity, the greater the amount of reactive oxygen species formed in the cell.

### 2.5. Animals

In the in vivo studies, male C57Bl/6 mice (age 9–10 weeks, weight 23–26 g) (*n* = 131), purchased from the “Stolbovaya” breeding facility (Scientific Center of Biomedical Technologies of the Federal Medical Biological Agency of Russia), were used. The animals were housed under standard laboratory conditions (temperature 20–22 °C, relative humidity 40–60%, 12 h light/dark cycle) with free access to food and filtered water. All procedures were performed in strict accordance with European Directive 2010/63/EU on the protection of animals used for scientific purposes. The study protocol was reviewed and approved by the Biomedical Research Ethics Committee of Ogarev Mordovia State University on 30.10.2025, meeting protocol No. 1.

### 2.6. In Vivo Study of MNP Biodegradation

The biodegradation of nanoparticles (Fe@OA, ZnMn@OA, Fe@SiO_2_ and ZnMn@SiO_2_) at the injection site was assessed based on the magnetization of the samples by vibrating sample magnetometry using an EZ11 magnetometer (Microsense Inc., USA) at 25 °C. Muscle tissue of the hind limb was collected on days 2 and 90 after intramuscular injection of MNPs at a dose of 256 mg/kg immediately after anesthesia of the animals with Zoletil (Virbac, Carros, France) and Rometar (Bioveta, Ivanovice na Hané, Czech Republic), followed by euthanasia by cervical dislocation. The obtained biomaterial was frozen at −80 °C and then homogenized until uniform. Standardized homogenate samples weighing 0.2 g were placed in plastic containers and dried to constant weight.

### 2.7. Antitumor Activity of Standalone Magnetic Hyperthermia (MHT) and Combination Therapy (MHT + Cisplatin)

The antitumor efficacy study was performed using a syngeneic model of solid Lewis lung carcinoma (LLC). The cell line was obtained from the Laboratory of the Bioresource Collection of Cell Lines and Primary Tumors of the N. N. Blokhin National Medical Research Center of Oncology (Moscow, Russia). For modeling tumor growth, donor material from the first in vivo passage was used. The tissue excised under aseptic conditions was minced and resuspended in Medium 199 containing an antibiotic. Transplantation was performed by subcutaneous injection of 50 µL of the resulting cell suspension at a concentration of 1 × 108 cells/mL into the lateral region of the left thigh. All invasive procedures, including implantation, and euthanasia were performed under general anesthesia with Zoletil (Virbac, Carros, France) and Rometar (Bioveta, Ivanovice na Hané, Czech Republic).

#### 2.7.1. Experimental Groups for Magnetic Hyperthermia (MHT) as a Standalone Treatment Modality and in Combination Therapy with Cisplatin

The experimental design followed a two-stage approach: at the first stage, the antitumor efficacy of four types of MNPs was comparatively evaluated under MHT conditions as a standalone treatment modality, which made it possible to select the most promising composition for the second stage of the study. At the second stage, the ability of MHT using the selected composition to enhance the antitumor effect of cisplatin was evaluated. This approach makes it possible not only to identify the most effective MNP configuration for local magnetic hyperthermia, but also to assess the feasibility of using MHT as a standalone treatment modality or as an adjunctive approach to chemotherapy in the treatment of Lewis lung carcinoma.

Experimental treatment was started on day 7 after implantation of LLC tumor cells, when the tumor volume reached approximately 150 mm^3^. The mice were randomized and allocated into 5 groups (*n* = 5 in each group for each experiment). Preliminary experiments provided no data indicating intrinsic toxicity of the MNP suspensions at the doses used, or an independent effect of AMF under the selected exposure parameters. Therefore, separate “MNPs without AMF” and “AMF without MNPs” groups were not included in the present study design.

In the first experiment (MHT as a standalone treatment modality), the antitumor efficacy of four types of MNPs was evaluated. For this purpose, 120 µL of the MNP suspension (154 mg/mL, corresponding to a dose of 800 mg/kg with respect to the magnetic material) was administered intratumorally in equal portions into 5 tumor sites 10 min before the start of therapy. Then, the animals injected with the corresponding MNPs were exposed to AMF for 30 min. The procedure was performed twice at an interval of 24 h.

In the second experiment (combination therapy, MHT + cisplatin), in the groups receiving chemotherapy alone, cisplatin was administered intraperitoneally on days 7, 9, and 11 after tumor-cell implantation so that the total course dose was 9 or 18 mg/kg. In the combination therapy groups, cisplatin was given 1 h before the first MHT session on day 7. 10 min before AMF exposure, ZnMn@OA was administered intratumorally in equal portions into 5 tumor sites. The mice were then exposed to AMF for 30 min twice, with a 24 h interval. Subsequent cisplatin injections in the combination therapy groups were performed on days 9 and 11.

Cisplatin was diluted in physiological saline (0.9% NaCl) to the required concentration and administered intraperitoneally in a volume of 0.5 mL. The experimental design is presented in Table 1.

#### 2.7.2. Magnetic Hyperthermia

Local magnetic hyperthermia of the tumor nodule was induced using an alternating magnetic field (100 kHz, 8 kA/m) generated by a Pharmag device (AMT&C Group, Troitsk, Russia) [38,41]. To exclude contact heating from the device components, the inductor was protected with a fluoroplastic shield. Animals were placed inside a thermally insulated coil with the tumor nodule positioned in the homogeneous-field region. Before the therapeutic experiments, the tumor surface temperature recorded by a thermal imaging camera was compared with the intratumoral temperature measured with a thermocouple. The intratumoral temperature was 1.4 ± 0.2 °C higher than the surface temperature. Heating was monitored using a Seek Thermal Compact PRO thermal imaging camera (Santa Barbara, CA, USA), which provided continuous recording of the surface temperature of the tumor and surrounding tissues. Therefore, the tumor surface temperature was maintained in the range of 44–45 °C, corresponding to an intratumoral temperature of 45–46 °C. The temperature was regulated throughout the therapy session by dynamically changing the magnetic field strength in the range from 6 to 8 kA/m.

#### 2.7.3. Chemotherapy

Cisplatin (Pharmasyntez-Nord JSC, Moscow, Russia) was used as a chemotherapeutic agent, a widely used platinum drug with a DNA-crosslinking mechanism of action used in the treatment of solid tumors, including lung cancer [44]. Cisplatin is also used in studies on the Lewis lung carcinoma model, including for evaluating the efficacy of combined antitumor approaches [15,45,46,47,48]. In the present study, cisplatin was administered at cumulative doses of 9 and 18 mg/kg. The dose of 18 mg/kg (6 mg/kg × 3) was considered a high-dose regimen close to the maximum tolerated dose burden for mice [49]. The dose of 9 mg/kg (3 mg/kg × 3) was considered a moderate regimen.

#### 2.7.4. Assessment of Tumor Growth and Survival

Tumor nodule volume was measured using a digital caliper on the day therapy was initiated and every 48 h thereafter. Tumor nodule volume was calculated using the formula V = (π × a × b^2^)/6, where (a) is the largest and (b) is the smallest tumor diameter. Tumor growth inhibition (TGI, %) was assessed using the formula: %TGI = 100 − ((V_t1_ − V_t0_)/(V_c1_ − V_c0_) × 100%), where V_t0_ and V_c0_ are the mean tumor volumes (in mm^3^) in the experimental and control groups, respectively, on the day therapy was initiated; V_t1_ and V_c1_ are the mean tumor volumes in the corresponding groups at the time of measurement [4,50]. For the analysis of overall survival, animal death or achievement of humane endpoint criteria was recorded daily, and Kaplan–Meier curves were constructed. Mouse body weight was measured every 48 h from the start of the experiment, and the results were expressed as percentages of the initial body weight on day 1.

#### 2.7.5. Blood Analysis, Histological Examination, and Determination of the Metastasis Index

To assess the systemic effects of combination therapy and chemotherapy based on hematological and biochemical blood parameters, 12 additional groups of mice were formed (*n* = 3 per group and time point). The animals were subjected to deep anesthesia with Zoletil (Virbac, Carros, France) and Rometar (Bioveta, Ivanovice na Hané, Czech Republic), followed by euthanasia on days 3 and 7 after treatment initiation. Following euthanasia by cervical dislocation, the animals were decapitated, and blood released from the cervical vessels was immediately collected into heparin-containing tubes. All procedures were performed according to standard laboratory methods: the hematological profile was assessed using a URIT-5160 analyzer (Medical Electronic Group, Guilin, China), and biochemical analysis of blood plasma was performed using a FUJI DRI-CHEM 4000ie analyzer (FUJIFILM, Tokyo, Japan). To assess metastatic lung involvement, the lungs were excised after animal death, cleared of surrounding tissues, and weighed. The metastasis inhibition index (MII, %) was calculated using a modified formula with lung weight as an integral indicator of metastatic burden: MII, % = [(M_control_ − M_treated_)/(M_contro_l − M_intact_)] × 100%, where M_control_ is the mean lung weight in untreated tumor-bearing animals; M_treated_ is the mean lung weight in animals after therapy; M_intact_ is the mean lung weight in intact animals without tumors [51,52,53]. Gross examination and counting of visible metastatic foci on the lung surface was also performed [54]. The number of metastases was expressed as the mean ± standard deviation (Mean ± SD) for each experimental group. For histological examination, lung and tumor tissue samples were fixed in 10% neutral buffered formalin. The tissues were embedded in paraffin, and 9–10 μm-thick sections were prepared using a PFM Rotary 3003 rotary microtome (PMF Medical, Cologne, Germany). Deparaffinized sections were stained with hematoxylin and eosin. Histological analysis and photomicrography were performed using a Nikon Eclipse Ni SS light microscope equipped with a Nikon DS Fi2 (Nikon Corporation, Tokyo, Japan) digital camera. Laboratory reference intervals for the measured hematological and biochemical parameters in male C57Bl/6 mice are provided in Table S1.

### 2.8. Statistics

Quantitative data are presented as the mean ± standard deviation (Mean ± SD) for the results of physicochemical characterization and in vitro experiments. The results of in vivo experiments are expressed as the mean ± standard error of the mean (Mean ± SEM). Differences between two independent groups were assessed using the Mann–Whitney U test. For multiple-group comparisons, one-way ANOVA with Tukey’s post hoc test or the Kruskal–Wallis test with Dunn’s post hoc test was used, as appropriate. Survival was analyzed using the Kaplan–Meier method and log-rank test. Differences were considered statistically significant at *p* < 0.05. Statistical analysis was performed using SPSS Statistics 24 (IBM, Armonk, NY, USA) and GraphPad Prism 8.0.1.

## 3. Results

### 3.1. Characteristics of MNPs

In this study, highly concentrated aqueous suspensions of iron oxide nanoparticles (IONPs) and zinc-manganese ferrite (Zn_0.2_Mn_0.8_Fe_2_O_4_) were obtained. To ensure colloidal stability and improve biocompatibility, the surface of the magnetic cores was modified with two types of shells. The first type was a coating based on oleic acid and sodium oleate (Fe@OA and ZnMn@OA), whereas the second type was amino-functionalized silicon dioxide (Fe@SiO_2_ and ZnMn@SiO_2_).

**Figure 1:**
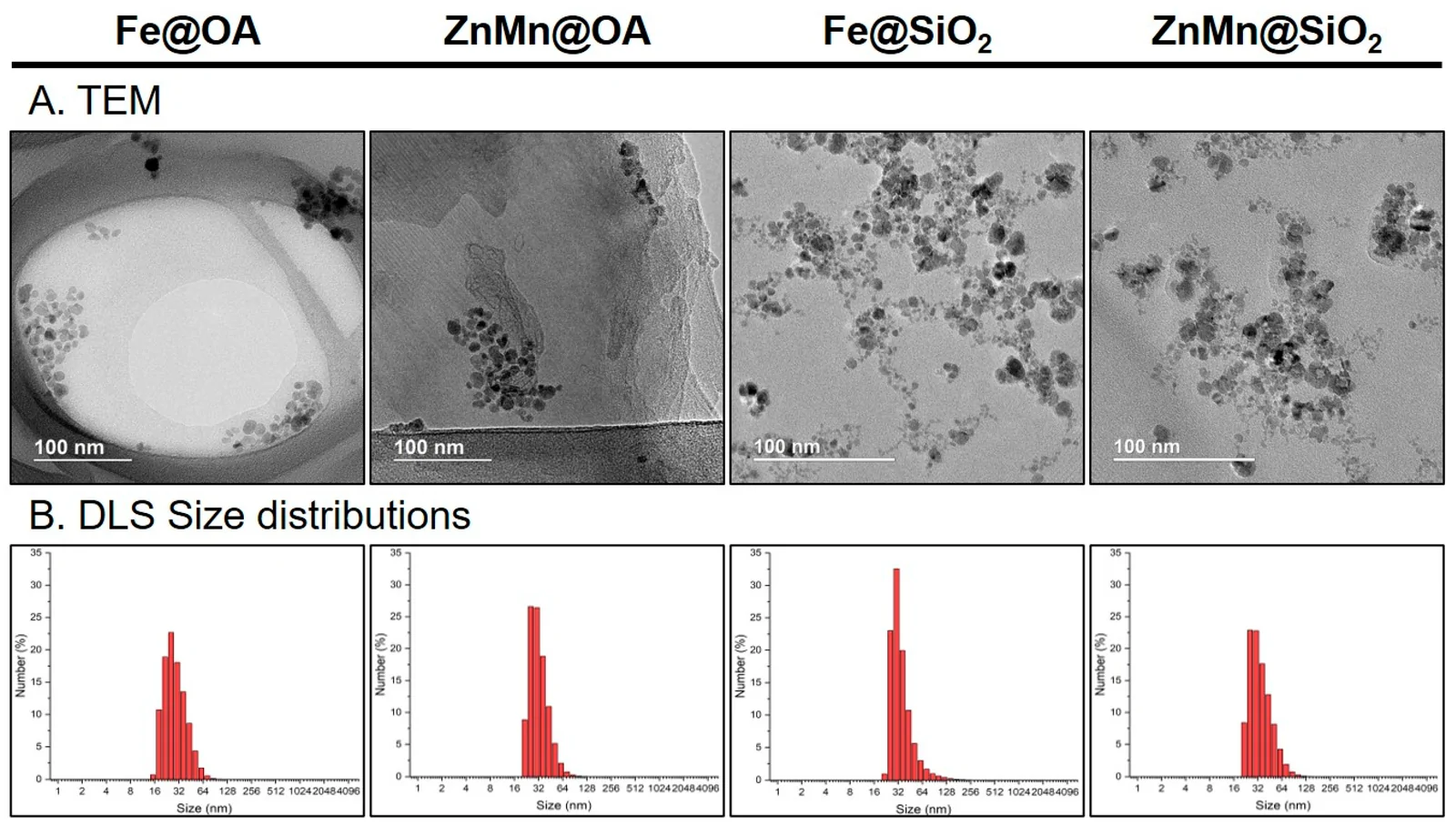
(**A**) TEM images and (**B**) DLS distribution of Fe@OA, ZnMn@OA, Fe@SiO_2_ and ZnMn@SiO_2_ magnetic nanoparticles.

As shown in the TEM images, Fe@OA, ZnMn@OA, Fe@SiO_2_ and ZnMn@SiO_2_ MNPs had a predominantly spherical or nearly spherical shape. Particle size distributions determined from TEM images are shown in Figure S5. The mean MNP size was as follows: Fe@OA—9 ± 1 nm; ZnMn@OA—9 ± 2 nm; Fe@SiO_2_—8 ± 2 nm; ZnMn@SiO_2_—9 ± 2 nm (Figure 1A).

The mean hydrodynamic diameter of the MNPs was as follows: Fe@OA—29 ± 10 nm, ZnMn@OA—31 ± 9 nm, Fe@SiO_2_—37 ± 17 nm, ZnMn@SiO_2_—35 ± 16 nm (Figure 1B).

**Figure 2:**
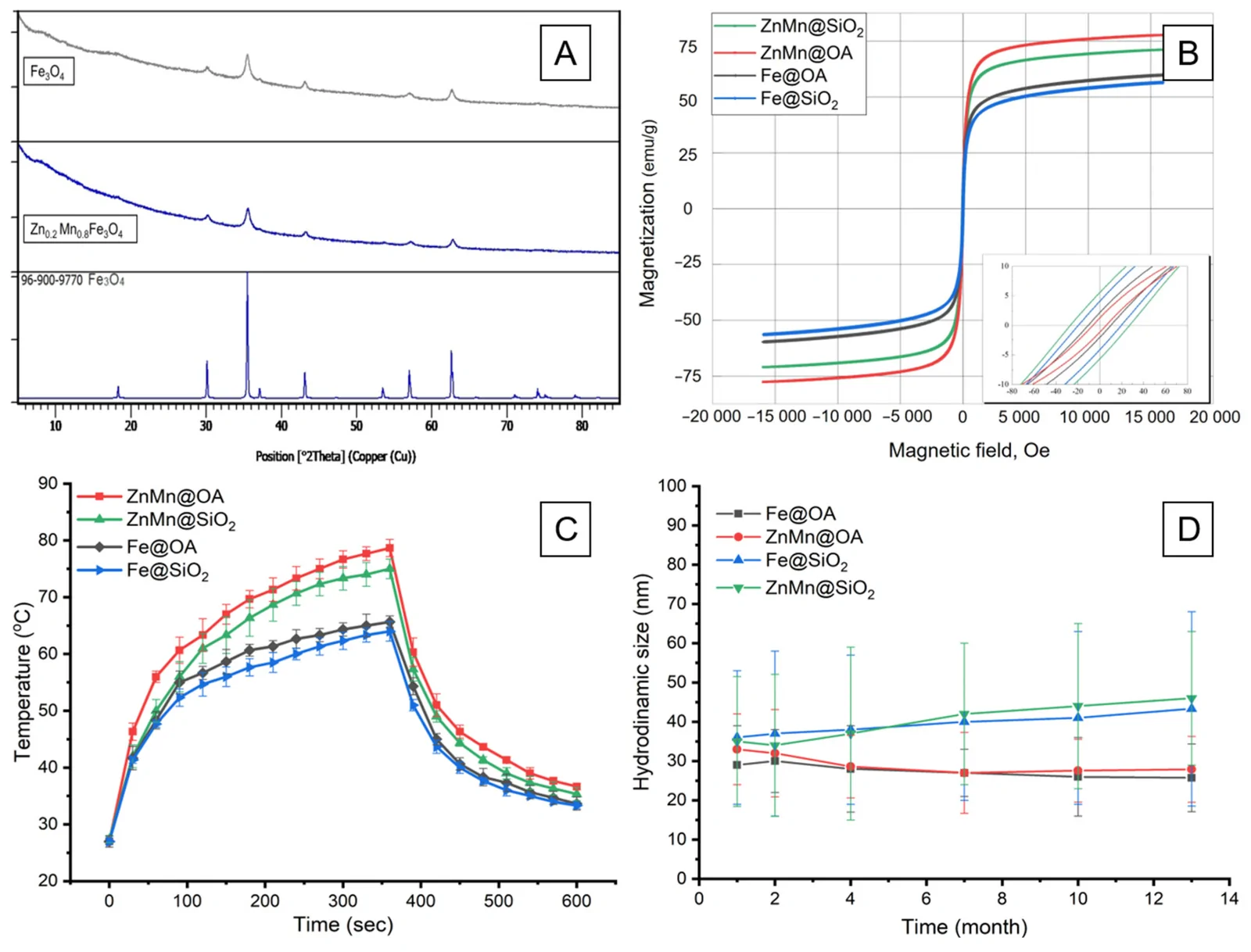
(**A**) X-ray diffraction patterns of Fe_3_O_4_ and Zn_0.2_Mn_0.8_Fe_2_O_4_, (**B**) saturation magnetization of nanoparticles in suspension, (**C**) heating and cooling curves of nanoparticles under exposure to an alternating magnetic field at f = 100 kHz and H = 8 kA/m, (**D**) hydrodynamic size of MNPs over 13 months for Fe@OA, ZnMn@OA, Fe@SiO_2_ and ZnMn@SiO_2_.

The polydispersity index (PDI) determined by DLS for all samples was in the range of 0.09–0.20 (Table 2). MNPs stabilized with oleic acid and sodium oleate were characterized by lower PDI values (by 1.8–1.9-fold) compared with the corresponding particles coated with silicon dioxide. X-ray diffraction (XRD) analysis (Figure 2A) showed that the obtained iron oxide nanoparticles (IONPs) and Zn_0.2_Mn_0.8_Fe_2_O_4_ nanoparticles exhibited diffraction patterns characteristic of a cubic spinel structure with the Fd-3m space group.

The magnetization curves (Figure 2B) confirmed the superparamagnetic state of the MNPs. Zinc-manganese ferrite nanoparticles exhibited higher saturation magnetization than IONPs (Table 2), which was attributed to their lower magnetocrystalline anisotropy [55].

The obtained suspensions efficiently converted the energy of an alternating magnetic field (f = 100 kHz, H = 8 kA/m) into heat. The peak temperature of the zinc-manganese ferrite-based suspensions was 15–20% higher than that of the IONP-based suspensions, regardless of the type of surface shell (Figure 2C). The SAR coefficient was calculated from the initial linear region of the average temperature dependence for each type of nanoparticle (Table 2). Figure 2D shows the results of the hydrodynamic diameter analysis of the nanoparticles over 13 months of storage, indicating that the particles retained their size. The SAR decreased by no more than 5% after 13 months for all MNPs.

### 3.2. Cytotoxicity and ROS Generation Assessment

**Figure 3:**
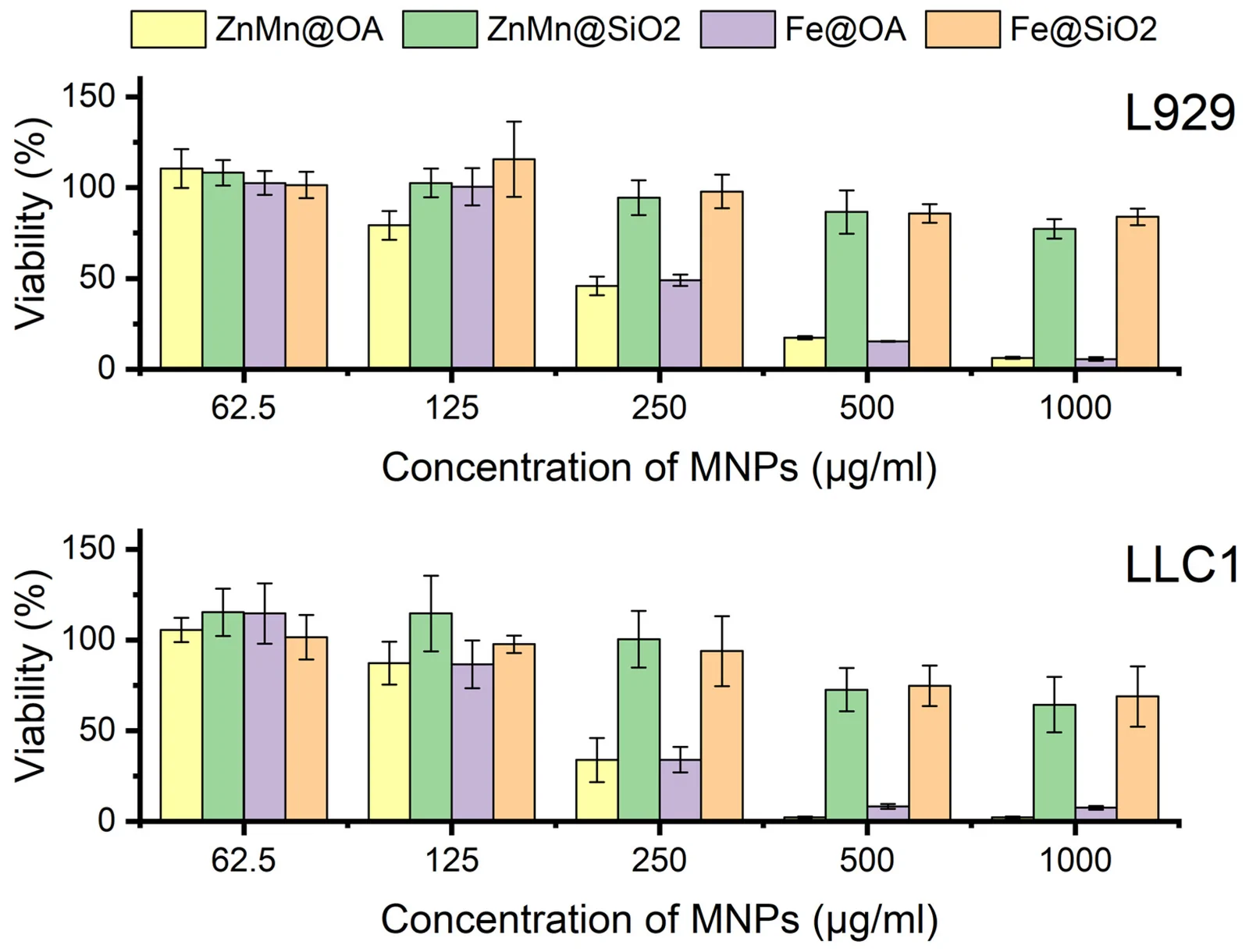
Viability of L929 and LLC1 cell lines after 24 h of incubation with Fe@OA, ZnMn@OA, Fe@SiO_2_, and ZnMn@SiO_2_ according to the MTT assay.

The results of the cytotoxicity assessment of Fe@OA, ZnMn@OA, Fe@SiO_2_ and ZnMn@SiO_2_ magnetic nanoparticles in Lewis lung carcinoma cells (LLC1) and mouse fibroblast cells (L929) are presented in Figure 3.

For silicon dioxide-stabilized nanoparticles, the viability of L929 and LLC1 cells did not decrease below 65% over the entire range of concentrations studied. Upon incubation with Fe@OA and ZnMn@OA MNPs, starting from a concentration of 125 μg/mL, L929 cells retained viability of at least 80%. LLC1 cells were found to be more sensitive to MNPs than L929 cells, regardless of the magnetic core material and shell.

For particles with a silicon dioxide shell (Fe@SiO_2_ and ZnMn@SiO_2_), IC50 values were not reached within the studied concentration range for either cell line. Fe@OA and ZnMn@OA nanoparticles demonstrated IC50 values of 253 and 249 μg/mL for L929 cells, whereas for LLC1 carcinoma cells, these values were 212 and 207 μg/mL, respectively.

**Figure 4:**
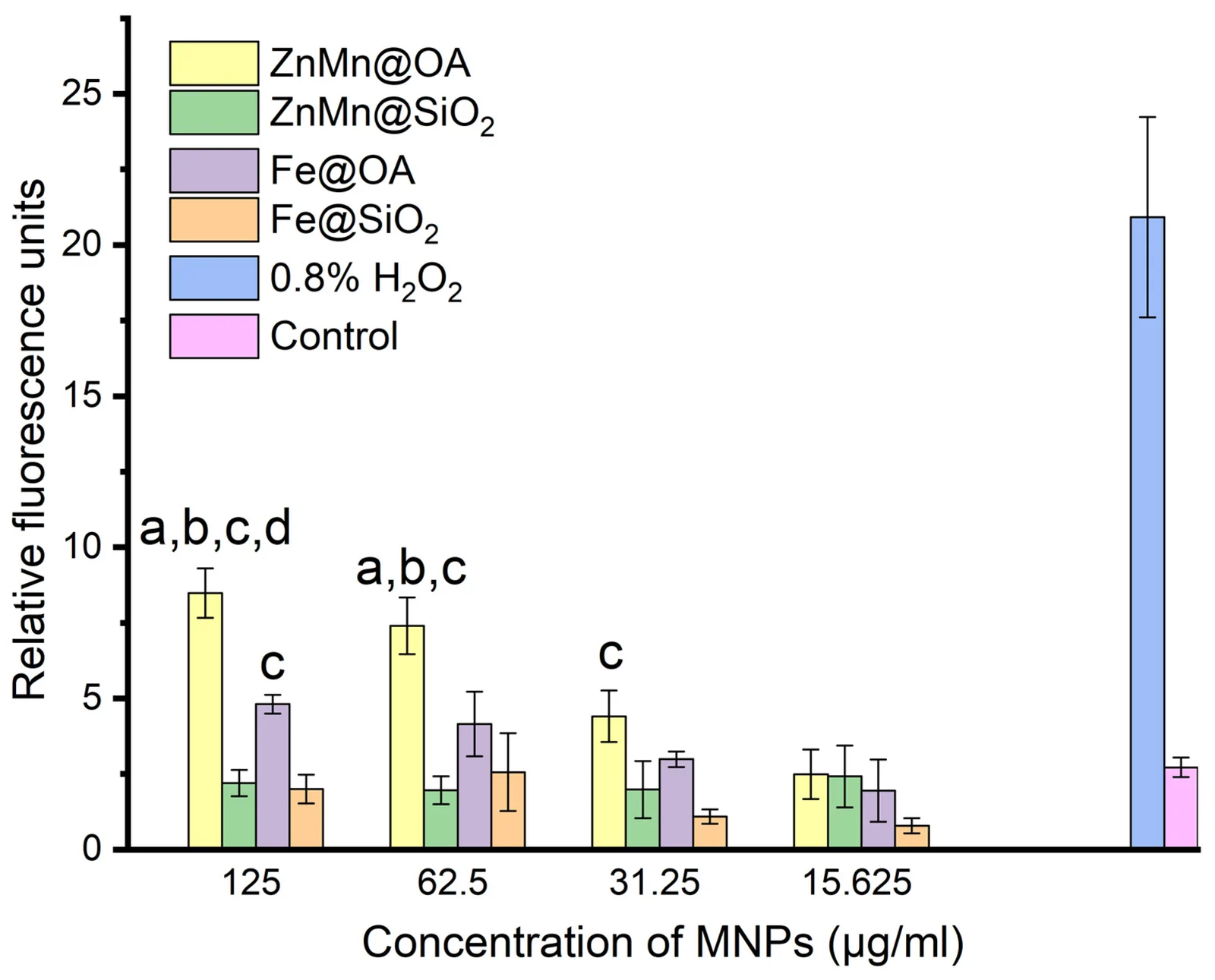
Results of the assessment of ROS generation in LLC1 cells after incubation with Fe@OA, ZnMn@OA, Fe@SiO_2_, and ZnMn@SiO_2_ nanoparticles. a—*p* < 0.05 compared with the control, b—*p* < 0.05 compared with ZnMn@SiO_2_, c—*p* < 0.05 compared with Fe@SiO_2_, d—*p* < 0.05 compared with Fe@OA.

The results of the assessment of intracellular reactive oxygen species production showed (Figure 4) that, upon incubation of LLC1 cells with silicon dioxide-stabilized MNPs (Fe@SiO_2_, ZnMn@SiO_2_) in the concentration range of 15–125 μg/mL, the level of the fluorescent signal did not differ statistically from the background control level.

The highest intensity of ROS generation among all tested samples was observed for ZnMn@OA. At a concentration of 125 μg/mL, this group differed significantly from the control and all other samples (ZnMn@SiO_2_, Fe@SiO_2_, and Fe@OA). At a concentration of 62.5 μg/mL, it differed significantly from the control, ZnMn@SiO_2_, and Fe@SiO_2_. At the same time, the level of ROS production in all experimental groups remained significantly lower than that in the positive control (0.8% H_2_O_2_).

### 3.3. Biodegradation of MNPs

**Figure 5:**
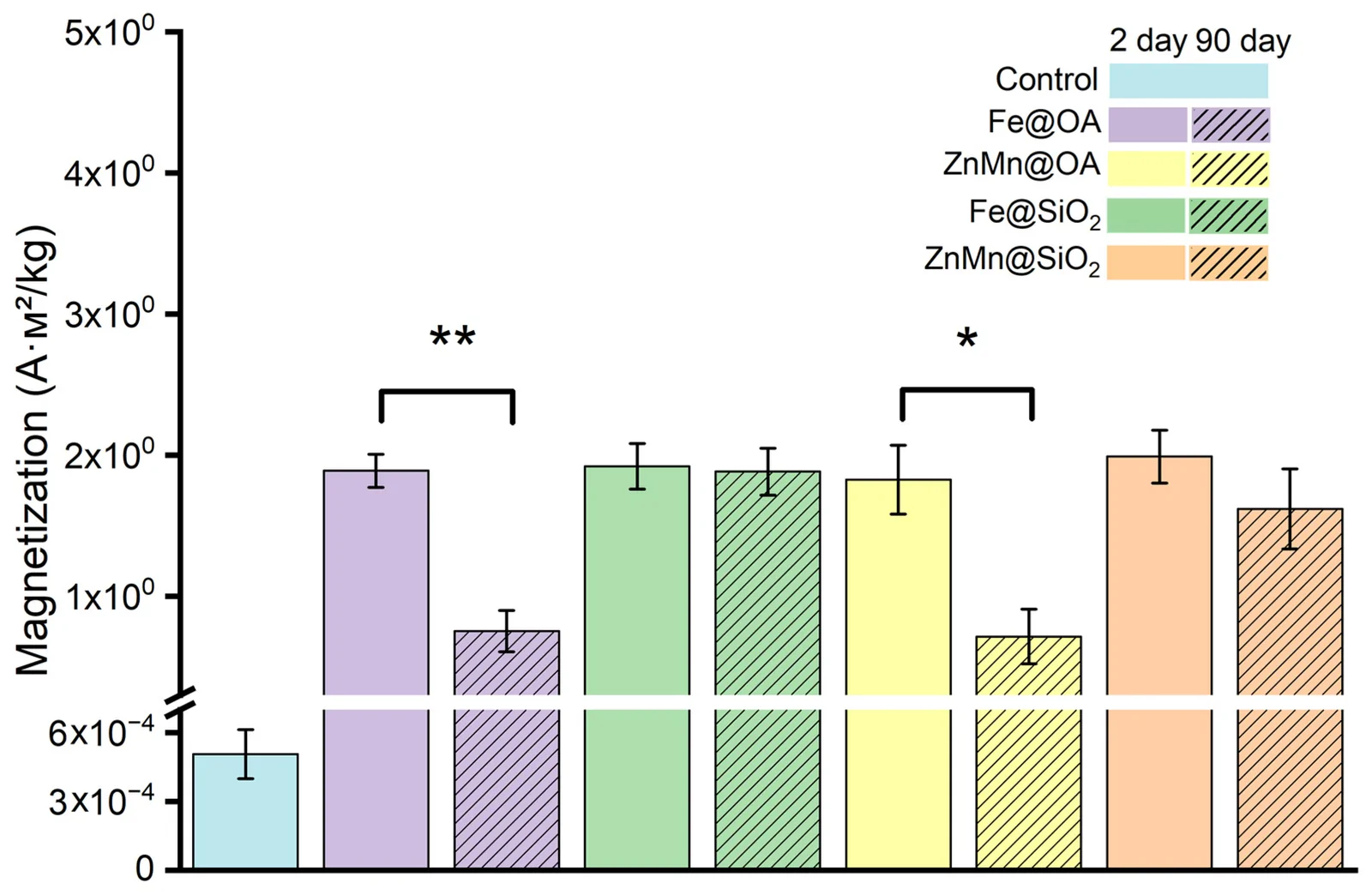
Magnetization, normalized to the mass of the muscle tissue sample, in the control and experimental groups (Fe@OA, ZnMn@OA, Fe@SiO_2_, and ZnMn@SiO_2_) on days 2 and 90 after intramuscular administration of MNPs at a dose of 256 mg/kg. Data are presented as M ± SEM (*n* = 5 per group and time point). Statistical significance of differences between observation time points (2 vs. 90 days): *—*p* < 0.05, **—*p* < 0.01.

The results of the magnetization analysis of tissue samples (Figure 5) showed the key influence of the chemical nature of the coating on MNP biodegradation. By day 90 after the administration of oleic acid-coated particles (Fe@OA and ZnMn@OA), the magnetic signal from muscle tissue had significantly decreased by 2.5-fold.

The decrease in magnetization indicates nanoparticle biotransformation under in vivo conditions. The oleic acid shell likely promotes degradation of the magnetic core material, which is consistent with previously described mechanisms of intracellular degradation of magnetic nanoparticles coated with bioorganic molecules [56,57].

In contrast, the magnetization of muscle tissue samples in the Fe@SiO_2_ and ZnMn@SiO_2_ MNP groups did not change after 90 days. The observed result can be explained by the fact that the inorganic SiO_2_ layer may act as a barrier protecting MNPs from dissolution in cells [58].

### 3.4. Antitumor Activity of Standalone MHT

The experiment assessing MHT as a standalone treatment was initiated on day 7 after transplantation of LLC tumor cells, when the tumor nodule volume reached approximately 150 mm^3^.

**Figure 6:**
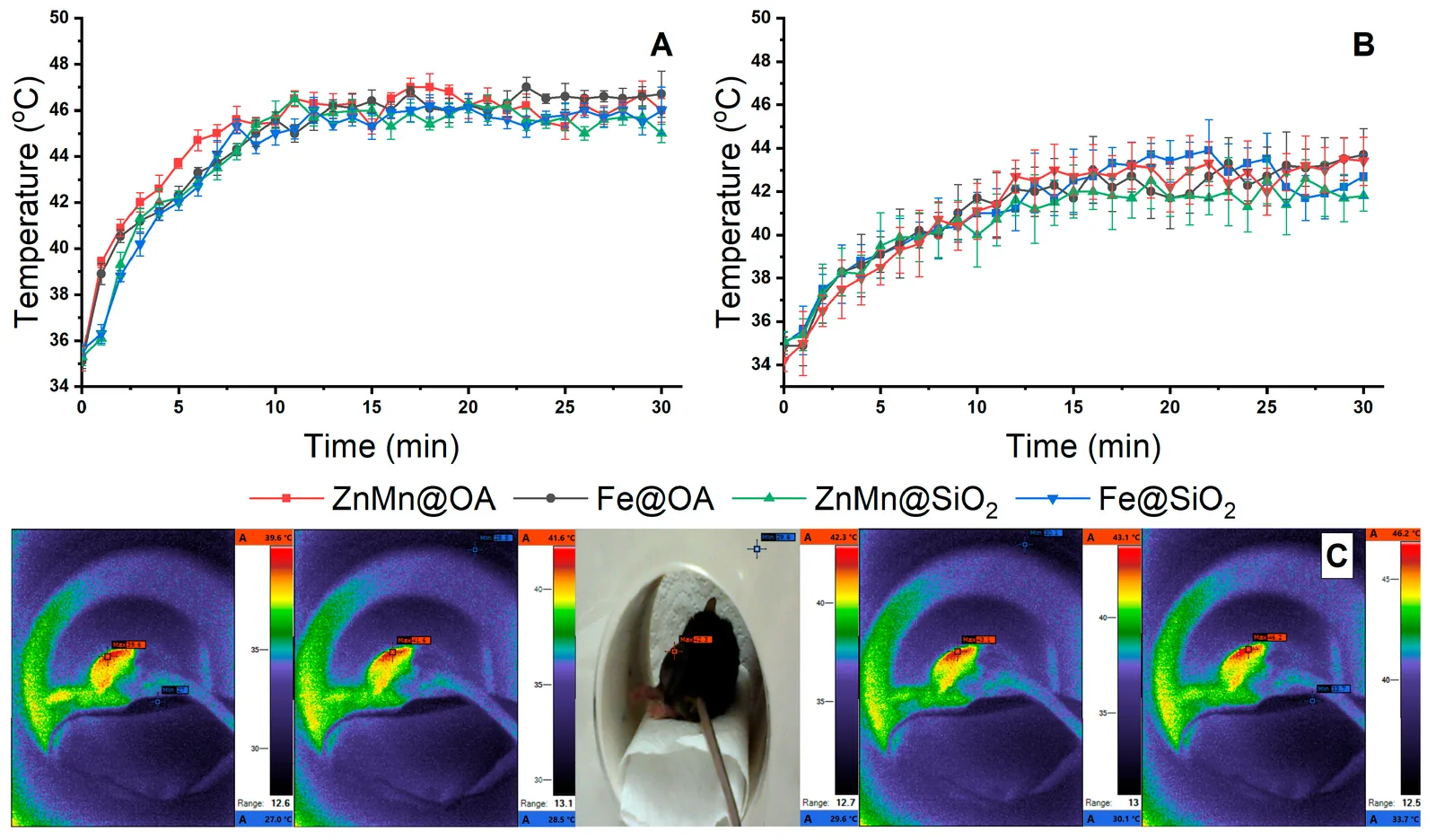
Temperature profile of the LLC tumor nodule in mice after intratumoral injection of magnetic nanoparticles (Fe@OA, ZnMn@OA, Fe@SiO_2_, and ZnMn@SiO_2_) and exposure to an alternating magnetic field at H = 8 kA/m and f = 100 kHz: (**A**,**B**) temperature changes in tumor tissue on the first and second days of therapy, respectively; (**C**) IR imaging of heat distribution in the tumor area.

After the start of the experiment, the target temperature of 45 °C was reached by 7 min for the ZnMn@OA suspension and by 8–9 min for the other compositions (Fe@OA, Fe@SiO_2_, and ZnMn@SiO_2_) (Figure 6A). Thus, the duration of hyperthermia within the therapeutic range was approximately 20 min.

During the repeated therapy session on day 2 (Figure 6B), a decrease in the thermal response was observed for all MNPs, with the maximum temperature reaching values in the range of 41–43 °C. A possible reason for the reduced heating efficiency during the second session may have been changes in the state of the tumor tissue after the first session, including the development of edema, necrosis, and areas of thermal damage [59]. An additional factor may have been the formation of a protein corona on the surface of the MNPs, followed by changes in their colloidal and aggregation state [60,61]. Aggregation and immobilization of the particles in tissues may restrict their Brownian relaxation, while enhanced dipole–dipole interactions may reduce heat generation efficiency in some systems [62,63,64].

**Figure 7:**
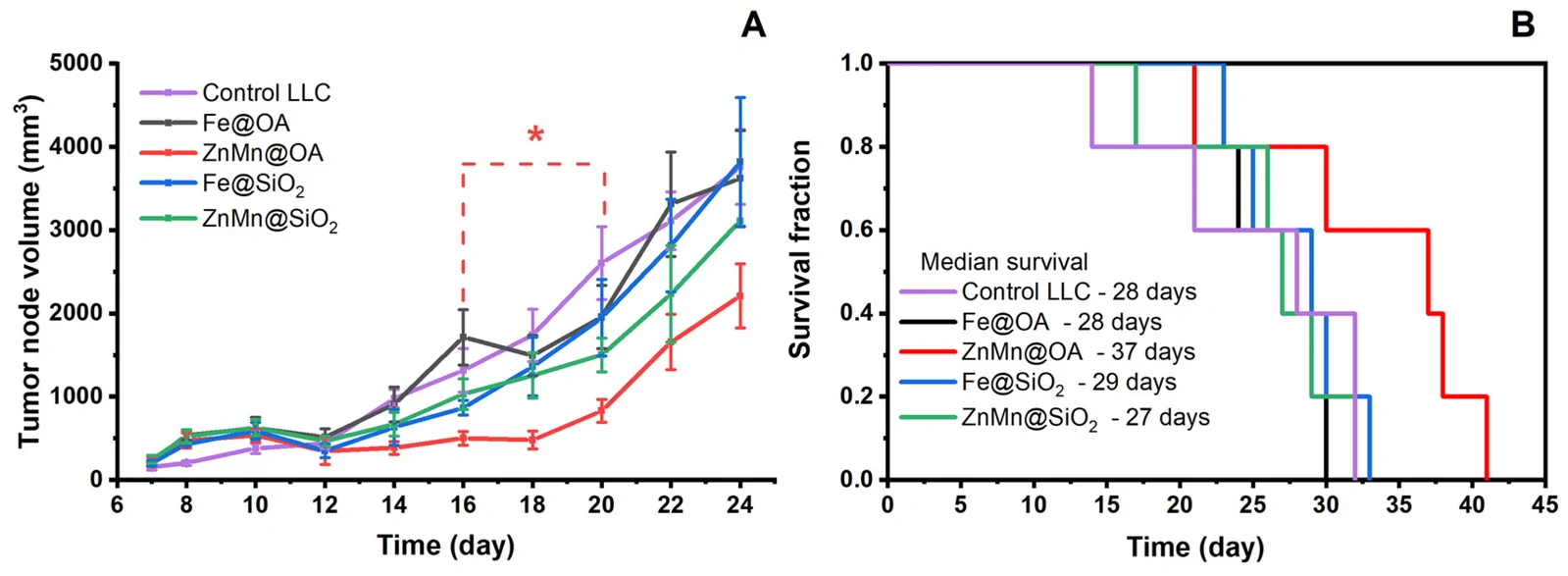
Indicators of the antitumor efficacy of standalone magnetic hyperthermia in the Lewis lung carcinoma model: (**A**) tumor node volume (mm^3^), data are presented as Mean ± SEM; (**B**) animal survival curves constructed by the Kaplan–Meier method. * *p* < 0.05 for the ZnMn@OA group compared with Control LLC.

Figure 7A shows the dynamics of tumor nodule growth. After the first and second magnetic hyperthermia sessions, edema developed in the tumor nodule area in all groups and persisted until day 12. This effect was likely a consequence of local heating of the tumor tissue accompanied by a transient inflammatory-edematous reaction. The most pronounced suppression of tumor growth was observed in the group of mice receiving MHT using ZnMn@OA. A statistically significant decrease in tumor volume versus Control LLC (*p* < 0.05) in the ZnMn@OA group was recorded from day 16 to day 20.

In this group, TGI values remained positive throughout the observation period, reaching a maximum of 83% on day 18 and remaining at 44–51% on days 22–24 (Figure S1). The maximum inhibitory effect was achieved on day 18 and was 83%. MHT using Fe@SiO_2_ and ZnMn@SiO_2_ was also accompanied by positive TGI values at individual observation time points; however, TGI values were below 50%. In the Fe@OA group, TGI values ranged from negative to moderately positive, indicating no sustained inhibition of tumor growth when this composition was used in the selected hyperthermia regimen (Figure S1).

Kaplan–Meier survival analysis (Figure 7B) showed that the maximum median survival (MS) was observed in the ZnMn@OA group and was 37 days, with the last animal in the group dying on day 41. In the control group, the median survival was 28 days. The use of the other nanoparticles did not increase median survival compared with the control: MS was 29 days for the Fe@SiO_2_ group, 28 days for Fe@OA, and 27 days for ZnMn@SiO_2_.

Among the studied samples, ZnMn@OA nanoparticles showed the most pronounced antitumor effect during MHT as a standalone treatment in the LLC model. Nevertheless, the use of physical treatment alone did not lead to a sustained decrease in tumor nodule volume or a statistically significant increase in survival versus control. Therefore, at the next stage of the study, ZnMn@OA MNPs were selected to evaluate the efficacy of a combined approach integrating magnetic hyperthermia and the chemotherapeutic agent cisplatin.

### 3.5. Combination Therapy (MHT + Cisplatin)

**Figure 8:**
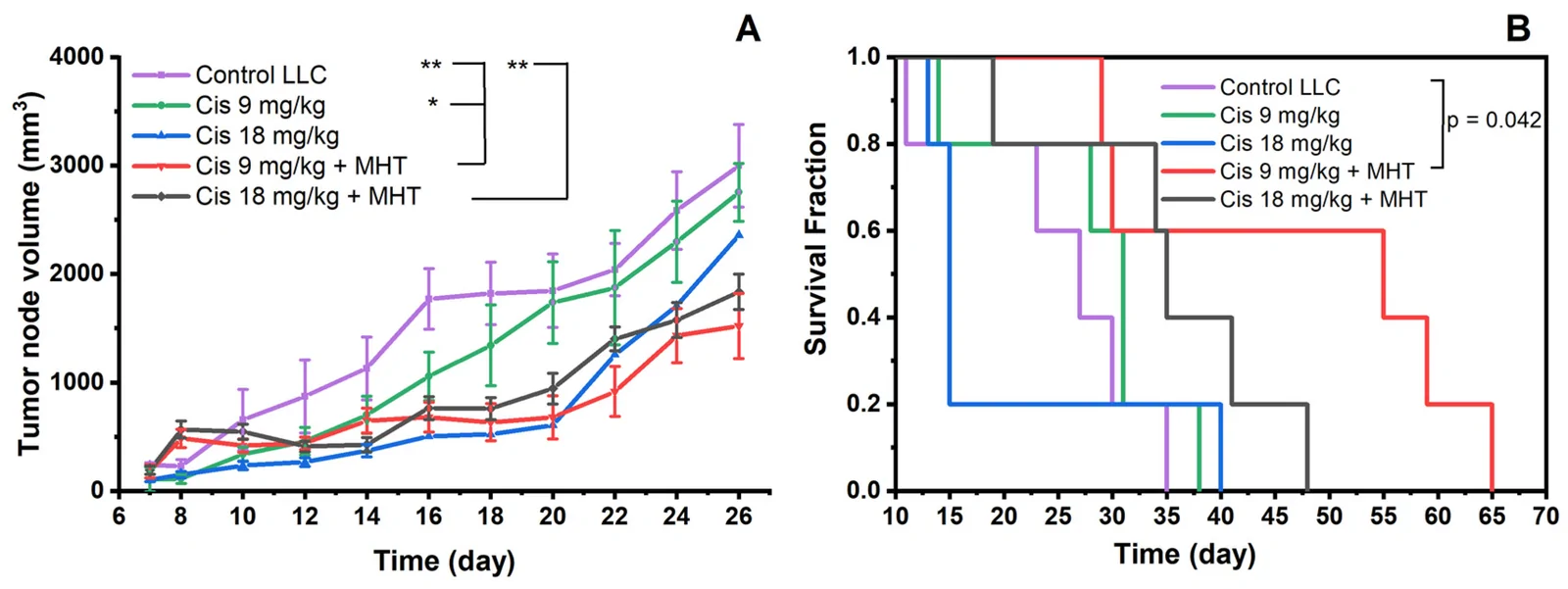
Indicators of the antitumor efficacy of different therapy regimens in the Lewis lung carcinoma model. (**A**) tumor node volume (mm^3^); (**B**) survival curves constructed by the Kaplan–Meier method. Data are presented as Mean ± SEM. * *p* < 0.05; ** *p* < 0.01; *n* = 5 per group.

The results of the study showed that cisplatin chemotherapy at a dose of 9 mg/kg as a standalone treatment did not provide pronounced suppression of tumor growth (Figure 8A). The dynamics of tumor nodule volume increases in this group were generally comparable to those in Control LLC. TGI values in the Cis 9 mg/kg group did not exceed 50%, decreasing from 43% on day 12 to 3.8% on day 26 (Figure S2). Chemotherapy with the higher dose of cisplatin (Cis 18 mg/kg) caused a pronounced early body weight loss (Figure S3) and death of most animals by day 15 of the experiment (Figure 8B). Therefore, after day 15, data on tumor growth dynamics (Figure 8A) in the Cis 18 mg/kg group are presented for only one surviving animal and were not used for intergroup statistical interpretation.

The most pronounced antitumor effect was achieved in the Cis 9 mg/kg + MHT combination therapy group. In this group, tumor node growth was slowed compared with Control LLC, significantly starting from day 16 of the experiment (Figure 8A). The TGI value in the Cis 9 mg/kg + MHT group remained positive throughout the entire observation period, reaching a maximum of 71% on day 18 and remaining at 46–59% by days 22–26 (Figure S2). Combination therapy with Cis 18 mg/kg + MHT was also accompanied by significant inhibition of tumor growth from day 16. However, based on the dynamics of tumor volume and TGI values, this effect was less sustained. After high TGI values at the early observation time points, the value decreased to 40% by day 26 (Figure S2). At the same time, no statistically significant differences in tumor volume were found between the two combination therapy groups.

Kaplan–Meier curve analysis showed that the highest median survival was recorded in the Cis 9 mg/kg + MHT group and was 55 days (Figure 8B). This was 2-fold higher than that in Control LLC, where median survival was 27 days, and 1.8-fold higher than that in the Cis 9 mg/kg group, where the median was 31 days. The difference between Control LLC and Cis 9 mg/kg + MHT was statistically significant according to the log-rank test (*p* = 0.042). Cisplatin chemotherapy alone did not prolong survival time: median survival was 31 days for Cis 9 mg/kg and 15 days for Cis 18 mg/kg. In the Cis 18 mg/kg + MHT group, median survival was 35 days.

The dynamics of animal body weight reflected differences in the tolerability of the investigated therapy regimens (Figure S3). In the Cis 18 mg/kg group, a sharp 40% decrease in body weight from baseline was observed, indicating systemic cisplatin toxicity at this dose. In the Cis 9 mg/kg group, body weight declined as well, but to a lesser extent (18% by day 18) and was followed by subsequent recovery. The combined use of Cis 18 mg/kg + MHT improved the body weight change profile compared with the corresponding chemotherapy alone. After a 19% decrease from baseline body weight by day 12, gradual recovery of body weight was observed. The most favorable body weight dynamics were observed in the Cis 9 mg/kg + MHT group, where animal body weight remained relatively stable throughout the entire observation period.

**Figure 9:**
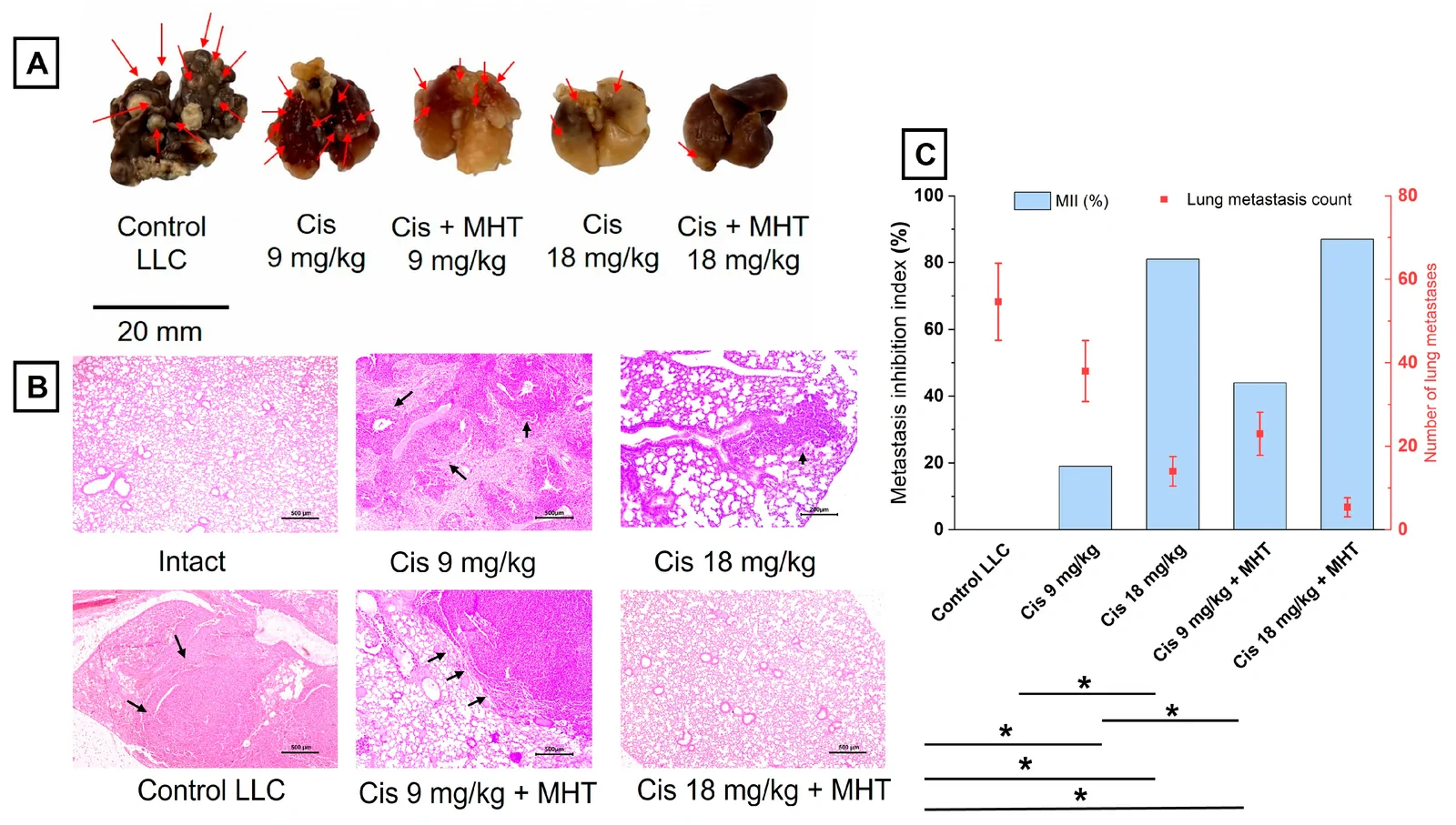
Assessment of the metastatic activity of LLC tumors after chemotherapy and combination therapy. (**A**) Representative macrophotographs of lungs with metastatic foci after different therapy regimens; red arrows indicate visible metastases. (**B**) Representative histological images of lung tissue, hematoxylin and eosin staining. Magnification ×40 and ×100, scale bars 500 and 200 μm, respectively. (**C**) Metastasis inhibition index (MII, %) and the number of lung metastases in the experimental groups. Statistical analysis was performed for the number of lung metastases, * *p* < 0.05 relative to the corresponding comparison groups. Arrows indicate the localization of metastatic foci.

Figure 9 presents the results of the assessment of metastatic lung involvement after different therapy regimens. Gross examination of the lungs showed pronounced metastatic involvement in the Control LLC group, where multiple metastatic foci were visualized on the lung surface as white or dark-gray granules (Figure 9A). In the groups receiving cisplatin, the number of visible metastases decreased in a dose-dependent manner. With Cis 9 mg/kg, metastatic foci in the form of granules persisted, although they were smaller in volume and diameter compared with the control. With Cis 18 mg/kg, the number of visible foci on the lung surface was noticeably lower, and the organ architecture was visually better preserved. Combination therapy with MHT also reduced the metastatic burden: fewer metastatic foci were visualized on the lung surface compared with Cis 9 mg/kg, with the most pronounced effect observed in the Cis 18 mg/kg + MHT group.

Histological examination of lung tissue confirmed the macroscopic findings (Figure 9B). In the Control LLC group, pronounced areas of tumor involvement of lung tissue were detected, whereas in the Cis 18 mg/kg and Cis 18 mg/kg + MHT groups, metastatic foci were the least pronounced, and the structure of the lung parenchyma was visually better preserved. In the Cis 9 mg/kg + MHT group, a reduction in metastatic involvement was also observed compared with Control LLC, although this effect was less pronounced than with the high dose of cisplatin. Quantitative analysis confirmed a reduction in metastatic burden under the influence of therapy (Figure 9C). In the control group, the number of visible lung metastases was 54.6 ± 9.2. In the Cis 9 mg/kg, Cis 18 mg/kg, Cis 9 mg/kg + MHT, and Cis 18 mg/kg + MHT groups, this value decreased to 38.0 ± 7.3, 14.0 ± 3.5, 23.0 ± 5.1, and 5.4 ± 2.3, respectively. Statistical analysis of the number of metastases showed a significant reduction in metastatic burden in the Cis 18 mg/kg, Cis 9 mg/kg + MHT, and Cis 18 mg/kg + MHT groups compared with Control LLC (*p* < 0.05). The metastasis inhibition index (MII%), calculated based on the change in lung weight relative to control and intact animals, was the highest in the Cis 18 mg/kg and Cis 18 mg/kg + MHT groups and was 81% and 87%, respectively. In the Cis 9 mg/kg and Cis 9 mg/kg + MHT groups, the MII values were 19% and 44%, respectively.

**Figure 10:**
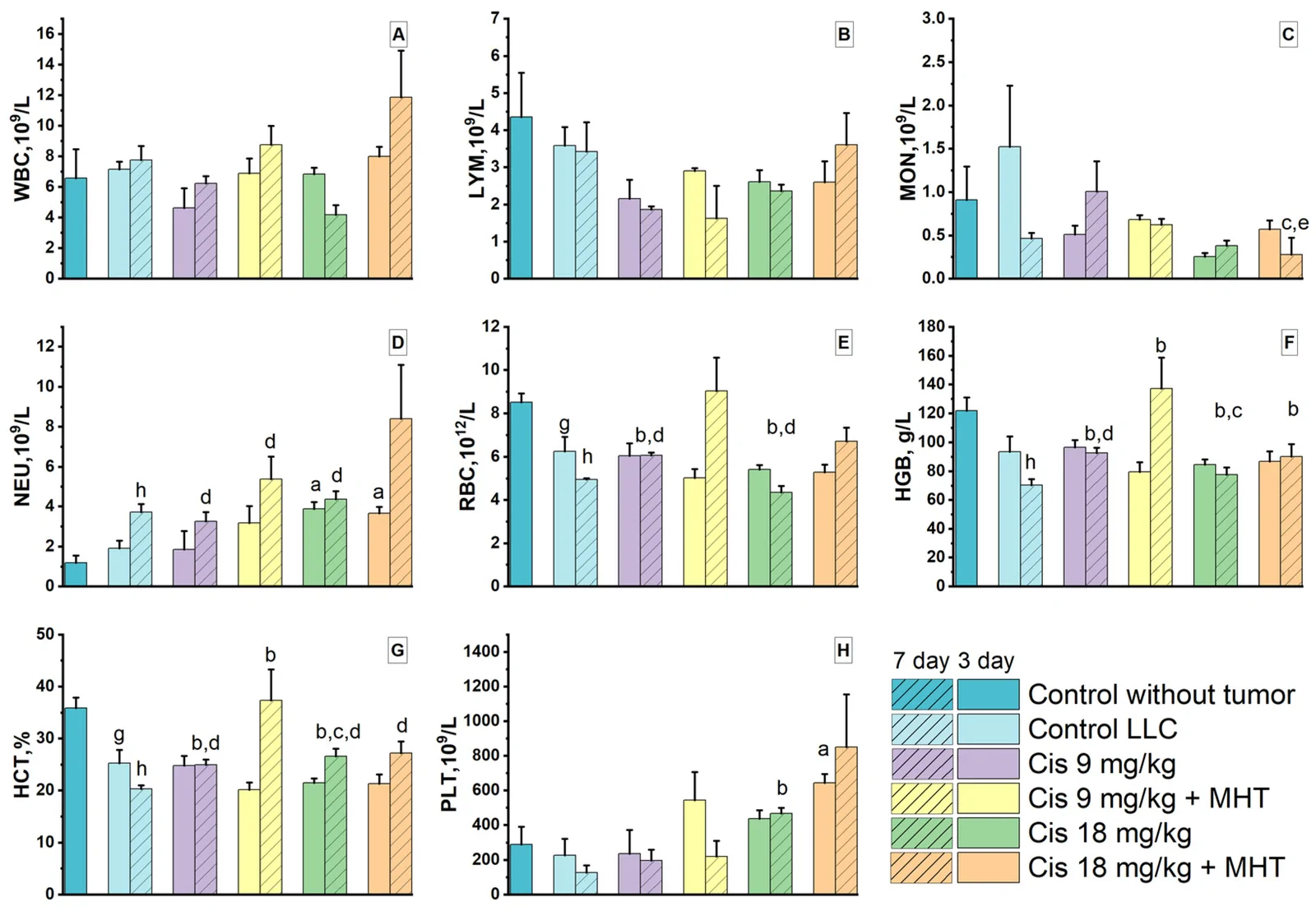
Effect of different therapy regimens on hematological blood parameters in mice with Lewis lung carcinoma on days 3 and 7 after therapy: (**A**) White blood cell count (WBC); (**B**) Lymphocyte count (LYM); (**C**) Monocyte count (MON); (**D**) Neutrophil count (NEU); (**E**) Red blood cell count (RBC); (**F**) Hemoglobin level (HGB); (**G**) Hematocrit (HCT); (**H**) Platelet count (PLT). Solid fill indicates data on day 3, hatched fill indicates data on day 7. Data are presented as mean ± SEM. Statistically significant differences (*p* < 0.05): (a, b) relative to the Control LLC group on days 3 and 7, respectively; (c) day 7 relative to day 3 within the experimental group; (d) relative to intact control (Control without tumor) on day 7; (e) comparison of combination therapy groups with different cisplatin doses on day 7; (g, h) Control LLC relative to intact control on days 3 and 7, respectively. (*n* = 3 per group and time point).

**Figure 11:**
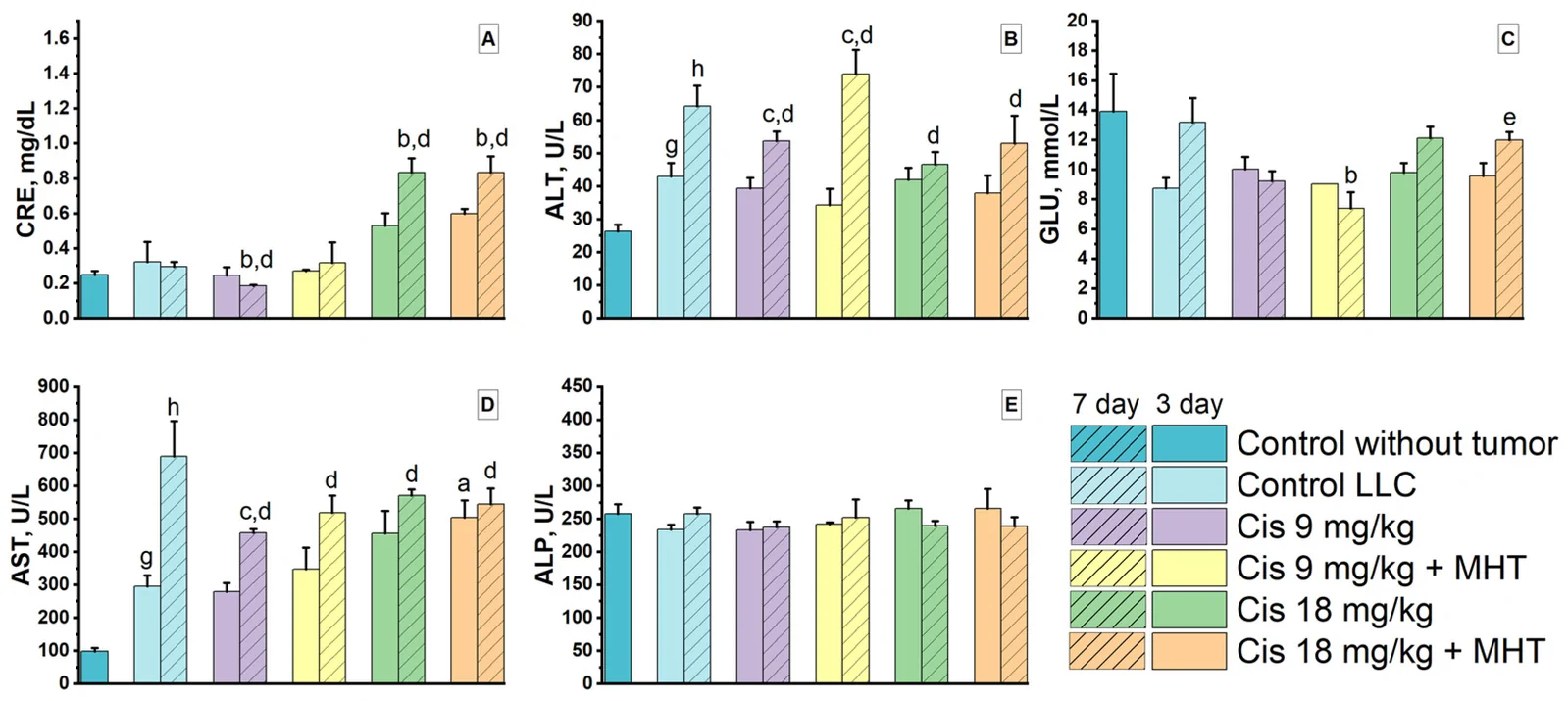
Effect of different therapy regimens on serum biochemical parameters in mice with Lewis lung carcinoma on days 3 and 7 after treatment: (**A**) creatinine level (CRE), (**B**) alanine aminotransferase level (ALT), (**C**) glucose level (GLU), (**D**) aspartate aminotransferase level (AST), and (**E**) alkaline phosphatase level (ALP). Solid fill indicates data on day 3, hatched fill indicates data on day 7. Data are presented as mean ± SEM. Statistically significant differences (*p* < 0.05): (a, b) relative to the Control LLC group on days 3 and 7, respectively; (c) day 7 relative to day 3 within the experimental group; (d) relative to intact control (Control without tumor) on day 7; (e) comparison of combination therapy groups with different cisplatin doses on day 7; (g, h) Control LLC relative to intact control on days 3 and 7, respectively. (*n* = 3 per group and time point).

The development of the tumor process in the Control LLC group was accompanied by changes in the cellular composition of blood and the biochemical profile (Figure 10 and Figure 11). Noticeable disturbances in hematological parameters were manifested by signs of tumor-associated anemia, including decreases in red blood cell count (RBC), hemoglobin level (HGB), and hematocrit (HCT) compared with intact tumor-free animals. Recovery of red blood cell parameters was observed in the Cis 9 mg/kg + MHT group. On day 7, RBC, HGB, and HCT levels in this group reached values comparable to those of intact tumor-free animals and did not differ statistically significantly from the intact control. At the same time, HGB and HCT differed significantly from Control LLC, indicating alleviation of tumor-associated anemia when MHT was combined with cisplatin at 9 mg/kg.

Evaluation of leukocyte parameters revealed an increase in neutrophils (NEU) in the Control LLC group, which may reflect the inflammatory component of the tumor process. In the combination therapy groups, especially with Cis 18 mg/kg + MHT, increases in WBC, NEU, and PLT were observed on day 7, which may be associated with the response of the hematopoietic system to the therapeutic intervention and tumor tissue damage.

Biochemical analysis showed that tumor growth was accompanied by signs of impaired liver function (Figure 11). In the Control LLC group, ALT and AST activities were increased compared with the intact control on both days 3 and 7 (Figure 11B,D). Therapeutic interventions did not lead to complete normalization of transaminases, and on day 7, ALT and AST levels in most groups remained higher than those in intact animals.

Evaluation of creatinine levels revealed differences between the therapy regimens. In the Cis 9 mg/kg and Cis 9 mg/kg + MHT groups, CRE levels remained relatively stable and did not show a marked increase compared with Control LLC. In contrast, chemotherapy with cisplatin at a dose of 18 mg/kg was accompanied by signs of nephrotoxicity. In the Cis 18 mg/kg group, a significant increase in CRE relative to Control LLC and the intact control was observed on day 7. In the Cis 18 mg/kg + MHT group, CRE levels were also increased on day 7, indicating persistent renal load when a high dose of cisplatin was used even as part of combination therapy.

Thus, combination therapy with Cis 9 mg/kg + MHT was characterized by the most favorable systemic profile among the therapeutic groups. It contributed to the recovery of red blood cell parameters to a level close to that of the intact control and was not accompanied by a marked increase in creatinine levels. At the same time, regimens including cisplatin at a dose of 18 mg/kg were accompanied by signs of systemic toxicity, including body weight loss and increased CRE levels.

## 4. Discussion

Stable, highly concentrated aqueous suspensions of magnetic nanoparticles based on iron oxide (IONPs) and zinc–manganese ferrite (Zn_0.2_Mn_0.8_Fe_2_O_4_), stabilized with oleic acid/sodium oleate (OA) or amino-functionalized silicon dioxide (SiO_2_–NH_2_), were obtained. The combined physicochemical characteristics of the obtained nanoparticles met the main requirements for magnetic hyperthermia agents [18,65]. All four samples had comparable magnetic core sizes according to TEM. The hydrodynamic diameter of the particles ranged from 29 to 37 nm and, as expected, exceeded the size determined by TEM because DLS accounts not only for the magnetic core but also for the surface stabilizing layer, the solvation shell, and the association of individual particles [66,67]. At the same time, the Fe@OA and ZnMn@OA samples were characterized by lower PDI values than the corresponding SiO_2_–NH_2_-coated particles. PDI values of approximately 0.2 or lower are considered desirable for a number of pharmaceutical nanosystems, although the specific acceptability criteria depend on the particle type [68,69]. The obtained results are consistent with published data on the ability of oleic acid to limit MNP agglomeration and promote the formation of uniform dispersions [32,70,71]. Particles with a Zn_0.2_Mn_0.8_Fe_2_O_4_ core exhibited higher saturation magnetization and SAR values than the corresponding iron oxide-based samples (Table 2). These results are consistent with the concept that the magnetic and magnetothermal properties of ferrites can be purposefully optimized by modifying their cationic composition [23,24,25,55,72]. The preservation of the hydrodynamic diameter over 13 months and a decrease in SAR of no more than 5% further confirm the stability of the obtained suspensions during long-term storage.

The in vitro study showed that the chemical nature of the magnetic core did not affect cytotoxicity when the coating type was identical. Nanoparticles coated with SiO_2_-NH_2_ did not reduce the viability of the cell lines (L929 and LLC1) below 65% over the entire range of concentrations studied (62.5–1000 μg/mL). It is known that magnetic nanoparticles with a silicon dioxide-based shell exhibit high biocompatibility [73]. In contrast, the oleic acid coating is characterized by a greater ability to interact with lipids of cell membranes. Lipophilic fatty acid-based shells, including oleic, lauric, palmitic, and stearic acids, can affect cellular uptake of nanoparticles and the magnitude of the biological response [74,75].

It was established that OA-coated zinc–manganese ferrite nanoparticles induced ROS formation more strongly than OA-coated IONPs. This is probably due to the high redox potential of manganese. Its ability to change oxidation state accelerates electron transfer in Fenton reactions and reduces the activation energy of H_2_O_2_ decomposition [76]. As a consequence, a high degree of manganese doping correlates with an increase in catalytic efficiency.

The chemical nature of the coating affects the biodegradation of MNPs. Thus, oleic acid is a bioorganic compound of the fatty acid class and indirectly ensures the degradation of the magnetic material under in vivo conditions.

As a standalone treatment, magnetic hyperthermia (MHT) exerted a temporary antitumor effect. The use of ZnMn@SiO_2_, Fe@SiO_2_, and Fe@OA nanoparticles did not show significant antitumor activity. The best result among the standalone MHT groups was demonstrated by ZnMn@OA particles, which provided statistically significant inhibition of tumor growth from days 16 to 20. This is probably associated with both the combination of the higher magnetothermal efficiency of the Zn_0.2_Mn_0.8_Fe_2_O_4_ core and the distribution features of OA-stabilized nanoparticles in tumor tissue. Histological analysis with Perls staining (Figure S4) showed that MNPs with a SiO_2_-NH_2_ coating were predominantly retained as dense compact aggregates, whereas particles with an OA coating were distributed in a more fragmented and diffuse manner. In the areas of MNP accumulation, regions of destruction and coagulative changes in tumor tissue, corresponding to local thermal damage, were also visualized.

It should be noted that, in a previously conducted toxicity study, a single local intramuscular administration of the ZnMn@OA suspension at the maximum tested dose of 1020 mg/kg caused no mortality, pronounced intoxication and local irritant effects. Also, intratumoral administration of all types of MNPs has no anticancer effect. Therefore, the tumor-suppression observed in the present experiment was attributed primarily to magnetic hyperthermia and its combination with chemotherapy. Data on the acute toxicity of the investigated magnetic suspensions are presented in the Supporting Materials, Table S2.

The resumption of tumor growth can be explained by the high aggressiveness of this model, the limited nature of local physical exposure without systemic chemotherapy, and the possible development of compensatory stress responses, including thermotolerance and angiogenesis [77,78,79]. The absence of a statistically significant increase in survival, sustained inhibition of tumor growth, and effects on metastatic progression, together with 100% animal mortality, indicates the limited efficacy of MHT as a standalone treatment in the LLC model. Nevertheless, ZnMn@OA was selected as one of the most promising formulations for further evaluation in combination therapy.

Our results showed that combination therapy using magnetic hyperthermia treatment and cisplatin provides the greatest antitumor effect compared with both standalone MHT and standalone chemotherapy. This was manifested not only in the suppression of primary tumor node growth, but also in a reduction in metastatic lung involvement, as well as in an increase in the lifespan of the animals. However, the efficacy and tolerability of the therapy were dose-dependent.

Cisplatin at a dose of 18 mg/kg exhibited pronounced antitumor activity. Both when used alone and in combination with MHT, this dose of the drug reduced tumor growth and decreased the number of metastases in the lungs. In addition, the addition of MHT increased median survival from 15 to 35 days in the Cis 18 mg/kg and Cis 18 mg/kg + MHT groups, respectively. The increase in survival was probably associated with an enhancement of the overall antitumor effect of combination therapy, including a reduction in metastatic burden: the number of metastases was 5.4 ± 2.3 in the Cis 18 mg/kg + MHT group versus 14.0 ± 3.5 in the Cis 18 mg/kg group. Nevertheless, the use of cisplatin at a dose of 18 mg/kg was accompanied by pronounced systemic toxicity. This was evidenced by a decrease in body weight of up to 40% in the Cis 18 mg/kg group and up to 20% in the Cis 18 mg/kg + MHT group, as well as an increase in creatinine levels. These data indicate that, despite pronounced suppression of tumor growth and metastasis, the toxic effect of cisplatin remained the main factor limiting animal survival in both groups that received the drug at a dose of 18 mg/kg.

On the other hand, the combination of Cis 9 mg/kg + MHT provided the best therapeutic profile. Significant inhibition of tumor node growth was observed relative to the control and the chemotherapy-only group (Cis 9 mg/kg), and the number of lung metastases was also reduced. This combination made it possible to significantly increase median survival 2-fold, to 55 days compared with 27 days in the control group (*p* = 0.042). The different effect of the 9 mg/kg dose on survival during standalone chemotherapy and in combination with MHT may be associated both with synergistic enhancement of the antitumor effect and with recovery of red blood cell parameters against the background of magnetic fluid administration. Analysis of hematological and biochemical parameters confirmed that combination therapy with Cis 9 mg/kg + MHT did not exert a critical negative effect on liver and kidney functions. Moreover, it contributed to the alleviation of tumor-associated anemia.

The higher efficacy of combination therapy compared with standalone use of cisplatin at the same dose may be associated with several complementary mechanisms of action of hyperthermia and chemotherapy. In the work by Oei et al., it was shown that hyperthermia affects DNA repair pathways and may increase the sensitivity of tumor cells to DNA-damaging agents [14]. This was experimentally confirmed by Schaaf et al., who showed that hyperthermia enhances the effect of cisplatin by inhibiting the PARP1-dependent DNA damage response (PARP1, poly(ADP-ribose) polymerase 1, a DNA repair enzyme) and delaying the repair of DNA damage [80]. In addition, hyperthermia may enhance the effect of cisplatin through disruption of mitochondrial metabolism: Sukovas et al. showed that the combination of hyperthermia at 43 °C with cisplatin in OVCAR-3 cells more strongly reduced the activity and expression of GDH (glutamate dehydrogenase), suppressed mitochondrial respiration, and decreased cell viability compared with each treatment separately [81]. In addition, Helderman et al. showed in an in vitro HIPEC model that platinum-based drugs, including cisplatin, exhibit temperature-dependent synergy with hyperthermia, which was accompanied by increased intracellular accumulation of the drug, enhanced DNA damage, and apoptosis [82].

Thus, the obtained data allow MHT to be considered not only as a local method for enhancing the antitumor effect of cisplatin, but also as an approach capable of improving the overall therapeutic balance between efficacy and toxicity.

The limitations of the study are the relatively small size of the experimental groups: *n* = 5 per group for the assessment of antitumor efficacy and *n* = 3 per group for hematological and biochemical parameters. This number of mice was chosen in accordance with the ethical principle of reducing the number of animals (the minimum number of animals sufficient for statistical analysis). Formal blinding of the investigators was not applied due to the features of the experimental design and the lack of practical feasibility. In addition, antitumor efficacy was evaluated using only one Lewis lung carcinoma model, which limits the direct generalization of the obtained results to other tumor types.

## 5. Conclusions

This work is the first to investigate and demonstrate the antitumor efficacy of MHT using Zn_0.2_Mn_0.8_Fe_2_O_4_@OA nanoparticles, both alone and combined with chemotherapy. The conducted study showed that local magnetic hyperthermia using safe alternating magnetic fields (100 kHz, 8 kA/m) and Zn_0.2_Mn_0.8_Fe_2_O_4_@OA nanoparticles enhances the efficacy of systemic cisplatin therapy in a mouse model of Lewis lung carcinoma. Comparative in vivo screening showed that zinc-manganese ferrite is the most promising magnetic material for generating heat under safe field conditions, while the oleic acid stabilizing coating ensures nanoparticle biodegradation in tissues. Taken together, this allows the presented combined regimen to be considered a promising therapeutic option for the LLC tumor model, as it combines a pronounced antitumor effect, reduced metastatic involvement, and an acceptable systemic toxicity profile.

## Figures and Tables

**Table 1:** Experimental design for standalone magnetic hyperthermia (MHT) and combination therapy (MHT + cisplatin).

	Experimental Groups		
Therapy	№	Therapy Schemes	Group Name
**Standalone Magnetic Hyperthermia (MHT)**	1	Fe_3_O_4_@OA (800 mg/kg) + MHT	Fe@OA
	2	Zn_0.2_Mn_0.8_Fe_2_O_4_@OA (800 mg/kg) + MHT	ZnMn@OA
	3	Fe_3_O_4_@SiO_2_–NH_2_ (800 mg/kg) + MHT	Fe@SiO_2_
	4	Zn_0.2_Mn_0.8_Fe_2_O_4_@SiO_2_–NH_2_ (800 mg/kg) + MHT	ZnMn@SiO_2_
	5	Mice with tumor without treatment	Control LLC
**Combination therapy (MHT + Cisplatin)**	1	Cisplatin 9 mg/kg	Cis 9 mg/kg
	2	Cisplatin 18 mg/kg	Cis 18 mg/kg
	3	Zn_0.2_Mn_0.8_Fe_2_O_4_@OA (800 mg/kg) + MHT + Cisplatin 9 mg/kg	Cis 9 mg/kg + MHT
	4	Zn_0.2_Mn_0.8_Fe_2_O_4_@OA (800 mg/kg) + MHT + Cisplatin 18 mg/kg	Cis 18 mg/kg + MHT
	5	Mice with tumor without treatment	Control LLC

**Table 2:** Results of the characterization of MNPs.

	Hydrodynamic Diameter (DLS), nm	PDI (DLS)	Diameter (TEM), nm	ζ-Potential, mV	Saturation Magnetization, emu/g	SAR, W/g
Fe@OA	29 ± 10	0.107	9 ± 1	–65 ± 3	60 ± 2	12 ± 0.3
ZnMn@OA	31 ± 9	0.089	9 ± 2	–67.4 ± 5	76 ± 3	15 ± 0.2
Fe@SiO_2_	37 ± 17	0.205	8 ± 2	+46 ± 4	58 ± 1	11 ± 0.2
ZnMn@SiO_2_	35 ± 16	0.151	9 ± 2	+41 ± 4	72 ± 2	13 ± 0.2

## Data Availability

The original data presented in this study are included in the article/Supplementary Materials. Further inquiries may be directed to the corresponding authors.

## Supplementary Materials

The following supporting information can be downloaded at: https://www.mdpi.com/article/10.3390/pharmaceutics18081021/s1, Figure S1. Tumor growth inhibition index (TGI, %) after standalone magnetic hyperthermia using different magnetic nanoparticle compositions in the LLC model. Figure S2. Tumor growth inhibition index (TGI, %) after standalone cisplatin chemotherapy and combined MHT + cisplatin therapy in the LLC model. Figure S3. Body weight dynamics of LLC-bearing mice after standalone cisplatin chemotherapy and combined MHT + cisplatin therapy. Figure S4. Representative histological images of LLC tumor tissue after standalone MHT with different magnetic nanoparticle compositions. Perls staining shows intratumoral distribution of Fe@OA, ZnMn@OA, Fe@SiO_2_, and ZnMn@SiO_2_ nanoparticles. Figure S5. Particle size distributions of magnetic nanoparticles determined from TEM images: (A) Fe@OA, (B) ZnMn@OA, (C) Fe@SiO_2_, and (D) ZnMn@SiO_2_. Figure S6. Energy-dispersive X-ray spectroscopy (EDX) spectra of magnetic nanoparticles with a SiO_2_-NH_2_ coating: (A) Fe@SiO_2_ and (B) ZnMn@SiO_2_. Table S1. Laboratory reference intervals for hematological and biochemical blood parameters in male C57Bl/6 mice based on data from 27 animals. Table S2. Acute toxicity parameters of magnetic nanoparticle suspensions after intraperitoneal and intramuscular administration. Reference [83] is cited in Supplementary Materials.
