# Supplementary material for: Magnetic Hyperthermia via Zn0.2Mn0.8Fe2O4 Oleic Acid Nanoparticles Enhances Chemotherapy Efficacy in a Lewis Lung Carcinoma Model
Source: Pharmaceutics. 2026 Aug 17;18(8):1021. doi: 10.3390/pharmaceutics18081021 (PMC13516219; doi:10.3390/pharmaceutics18081021)
Supplement: Supplementary file 1 [file pharmaceutics-18-01021-s001.zip › pharmaceutics-4419241-supplementary.pdf]

Magnetic Hyperthermia via $Zn_{0.2}Mn_{0.8}Fe_2O_4$ Oleic Acid Nanoparticles Enhances Chemotherapy Efficacy in a Lewis Lung Carcinoma Model

Materials and Methods

For histological examination, muscle tissue samples were fixed in 10% neutral buffered formalin. The samples were dehydrated through a graded ethanol series, cleared in xylene, embedded in paraffin, and sectioned at a thickness of 9-10 µm using a PFM Rotary 3003 rotary microtome (PMF Medical, Germany). Perls' Prussian blue staining was performed to detect iron-containing nanoparticle deposits in muscle tissue. Briefly, deparaffinized sections were rehydrated through descending concentrations of ethanol to distilled water and incubated in freshly prepared Perls' reagent, consisting of equal volumes of 2% potassium ferrocyanide and 2% hydrochloric acid, for 30 min at room temperature. After incubation, the sections were thoroughly rinsed in distilled water, counterstained with hematoxylin and eosin, dehydrated through ascending concentrations of ethanol, cleared in xylene, and mounted with a coverslip. Histological specimens were examined using a Nikon Eclipse NI-SS light microscope (Nikon Corporation, Japan). Microphotographs were obtained using a Nikon DS-Fi2 camera attachment (Nikon Corporation, Japan) at ×40, ×100, and ×400 magnification.

Results

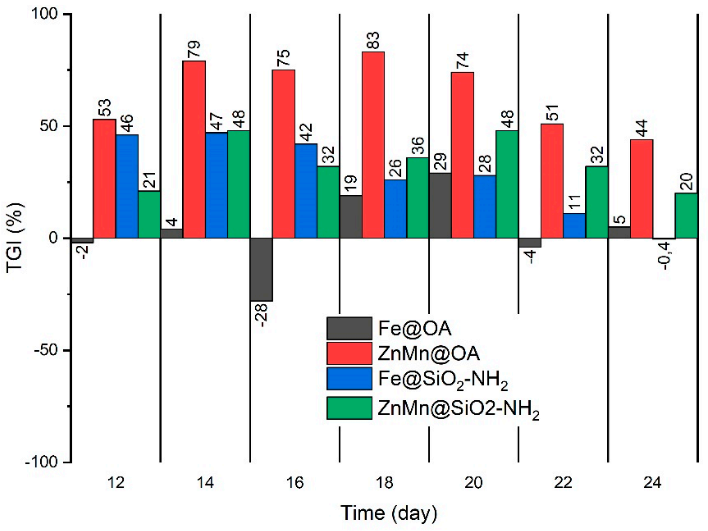

Figure S1. Tumor growth inhibition index (TGI, %) after stand-alone magnetic hyperthermia using different magnetic nanoparticle compositions in the LLC model.

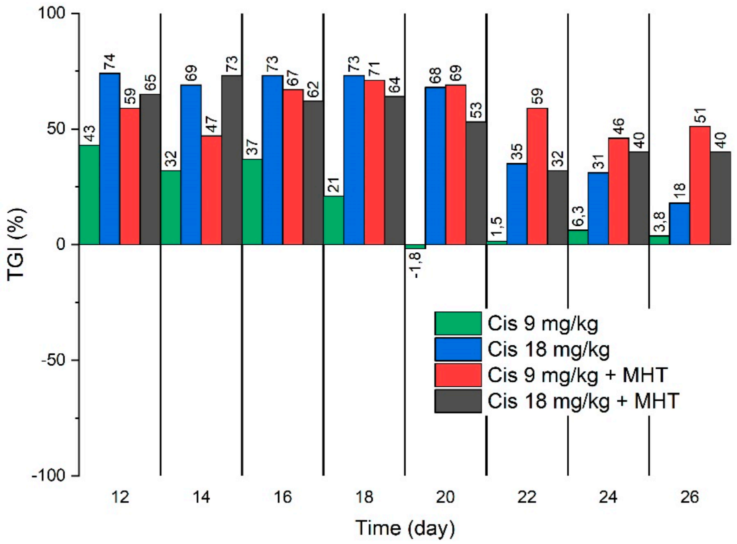

Figure S2. Tumor growth inhibition index (TGI, %) after stand-alone cisplatin chemotherapy and combined MHT + cisplatin therapy in the LLC model.

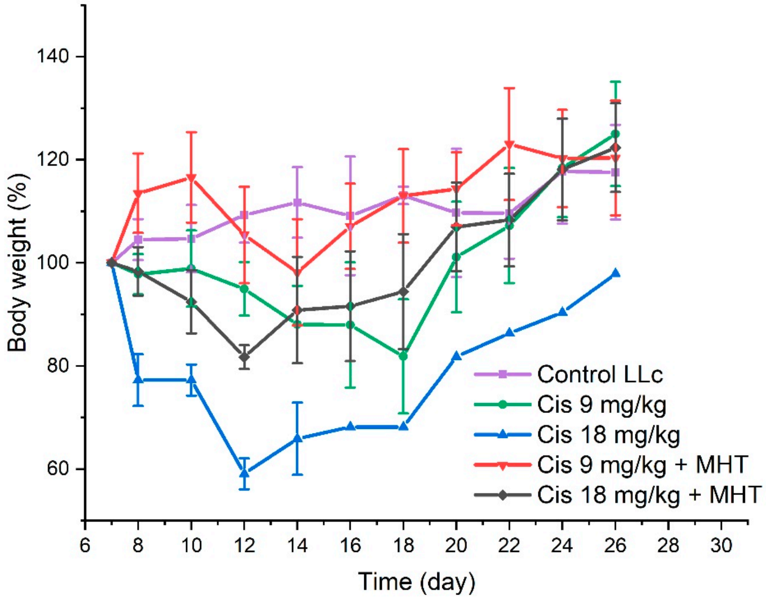

Figure S3. Body weight dynamics of LLC-bearing mice after stand-alone cisplatin chemotherapy and combined MHT + cisplatin therapy.

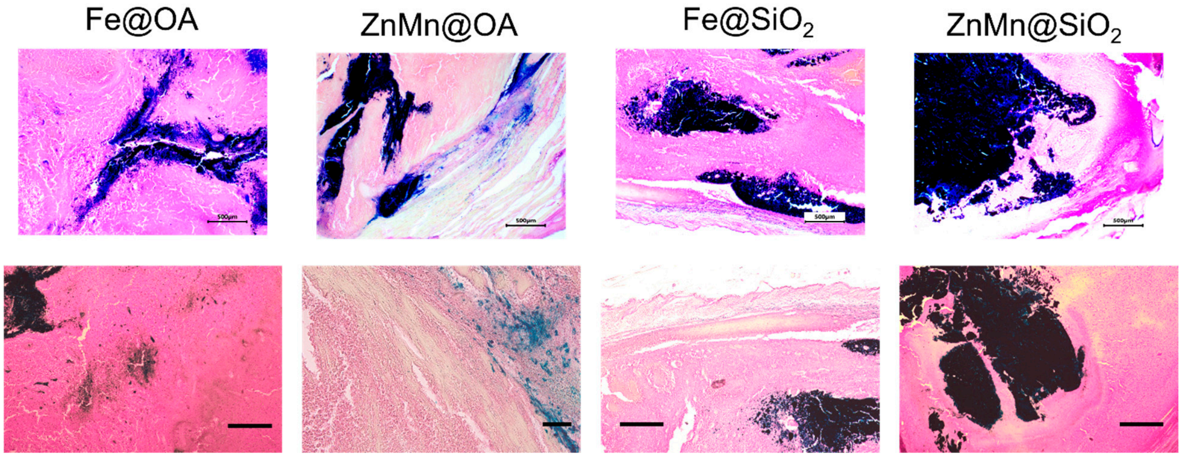

Figure S4. Representative histological images of LLC tumor tissue after stand-alone MHT with different magnetic nanoparticle compositions. Perls staining shows intratumoral distribution of Fe@OA, ZnMn@OA, Fe@SiO_2, and ZnMn@SiO_2 nanoparticles.

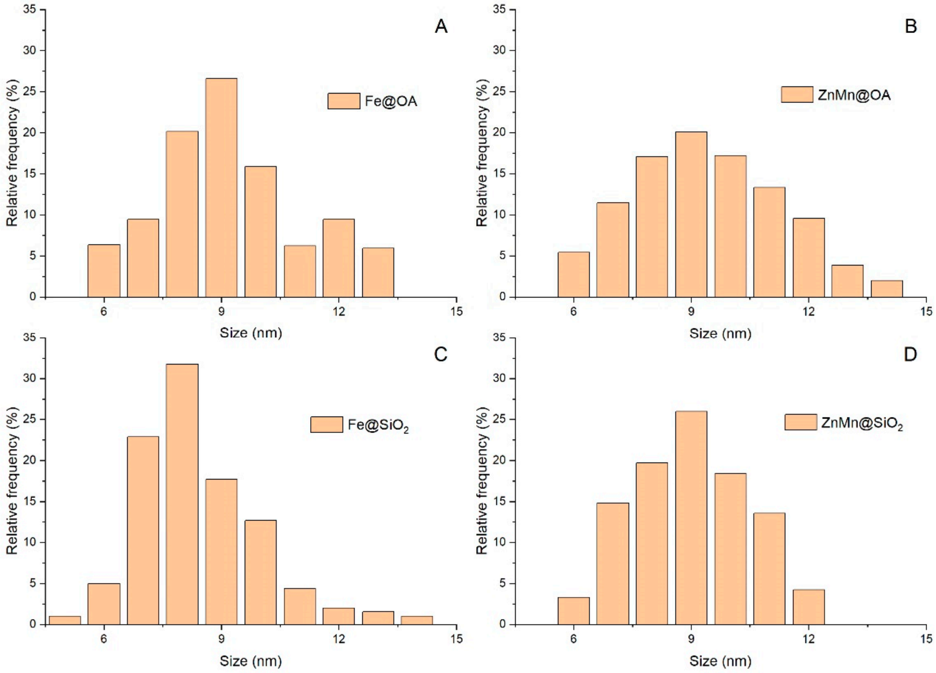

Figure S5. Particle size distributions of magnetic nanoparticles determined from TEM images: (A) Fe@OA, (B) ZnMn@OA, (C) Fe@SiO_2, and (D) ZnMn@SiO_2. Data are presented as relative frequency (%).

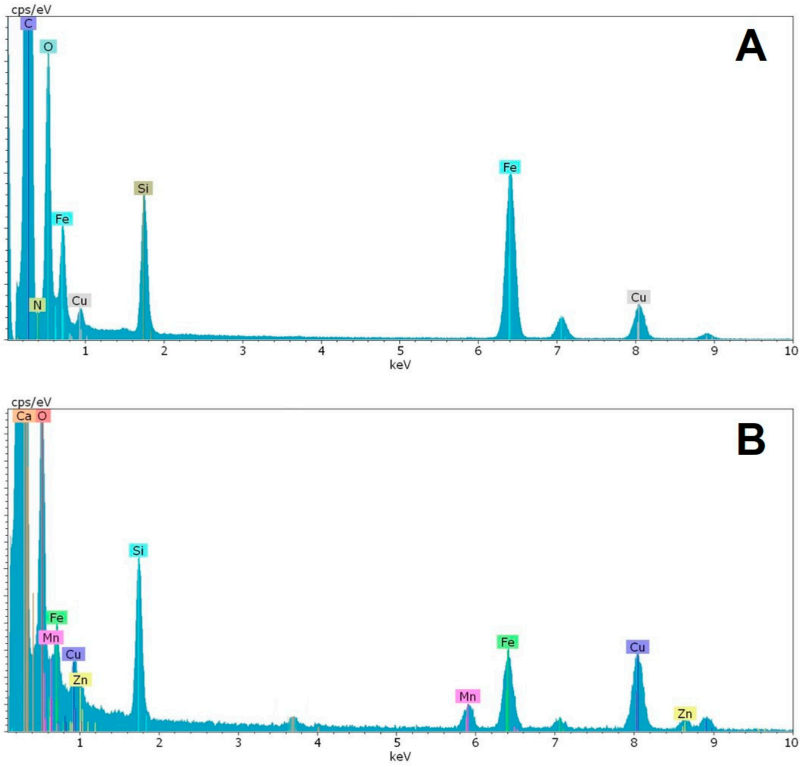

Figure S6. Energy-dispersive X-ray spectroscopy (EDX) spectra of magnetic nanoparticles with a SiO_2-NH_2 coating: (A) Fe@SiO_2 and (B) ZnMn@SiO_2. The Si signal confirms the presence of silicon in both samples; Cu signals originate from the TEM grid.

Table S1. Laboratory reference intervals for hematological and biochemical blood parameters in male C57Bl/6 mice based on data from 27 animals. Values are presented as the mean and the 2.5th–97.5th percentile interval.

Hematological blood parameters	
Parameters	Mean [2.5th–97.5th percentiles]
WBC,10^9/L	5.8 [2.4-10.8]
LYM,10^9/L	3.1 [1.1-6.0]
MON,10^9/L	0.8 [0.3-1.2]
NEU,10^9/L	1.9 [0.3-5.5]
RBC,10^{12}/L	7.2 [6.2-8.9]
HGB, g/L	116.4 [97.1-138.4]
HCT, %	32.6 [2.4-10.8]
PLT,109/L	344.0 [99.2-611.0]
Biochemical parameters	
CRE, mg/dL	0.27 [0.2-0.4]
ALT, U/L	25.3 [17.1-37.4]
GLU, mmol/L	12.2 [8.1-15.9]
AST, U/L	156.1 [91.2-188]
ALP, U/L	260.0 [226.0-313.6]

Table S2. Acute toxicity parameters of magnetic nanoparticle suspensions after intraperitoneal and intramuscular administration.

	LD50 after intraperitoneal administration, mg/kg (95% CI)	LD50 after intramuscular administration, mg/kg
Fe@OA	904 (635–1172)	>1020
ZnMn@OA	1145 (917–1372)	>1020
Fe@SiO2	Not reached within the tested dose range	>1020
ZnMn@SiO2	2755 (2333–3162)	>1020

Acute toxicity was evaluated after a single intraperitoneal or intramuscular administration of the magnetic nanoparticle suspensions. LD50 values after intraperitoneal administration were calculated from mortality data obtained during the 14-day observation period. After intramuscular administration, no mortality or pronounced signs of intoxication were observed at the maximum tested dose of 1020 mg/kg, which was limited by the maximum feasible administration volume of 0.2 mL; therefore, the LD50 was not reached for any of the suspensions. According to the classification proposed by Berezovskaya for acute toxicity after parenteral administration, all investigated suspensions may be classified as practically non-toxic or relatively harmless following intramuscular administration (Berezovskaya, I.V. Classification of Substances with Respect to Acute Toxicity for Parenteral Administration. Pharmaceutical Chemistry Journal 2003, 37, 139–141. DOI: 10.1023/A:1024586630954).
